# Multi-year evaluation and release of improved soybean lines for resistance to Phomopsis seed decay

**DOI:** 10.1371/journal.pone.0354166

**Published:** 2026-08-24

**Authors:** Shuxian Li, James R. Smith, Anne M. Gillen, Quentin D. Read

**Affiliations:** 1 United States Department of Agriculture, Agricultural Research Service (USDA, ARS), Crop Genetics Research Unit, Stoneville, Mississippi, United States of America; 2 USDA, ARS, Southeast Area Office, Raleigh, North Carolina, United States of America; Mustafa Kemal University: Hatay Mustafa Kemal Universitesi, TÜRKIYE

## Abstract

Soybean [*Glycine max (L.)* Merr.] is one of the most economically important oilseed crops grown in the world. Soybean diseases not only affect yield but can also affect seed quality resulting in significant economic losses. Phomopsis seed decay (PSD) causes poor seed quality and is one of the most damaging soybean seed diseases. *Diaporthe longicolla* (syn. *Phomopsis longicolla*) and other pathogens in the *Diaporthe*/*Phomopsis* complex are the cause of PSD. Developing and releasing PSD-resistant germplasm lines is an important step toward the development of resistant cultivars that can protect soybean from PSD seed damage. The objective of this study was to identify improved soybean lines with PSD resistance for the mid-southern U.S. after inoculation with *D. longicolla* in a 5-year delayed-harvest field trial. In this study, seed assays were conducted to evaluate a total of 266 soybean entries in field experiments with inoculation of *D. longicolla* from 2019 to 2023. Results from five years of testing showed that maturity group (MG) late III 65-414-132-1 (DS65-1) had significantly less infection (38.6%) than susceptible late III LG03-4561-14 (86.2%). Early MG IV lines 14119-211-10 and 10031-243-12 (released as DS31-243) had statistically lower levels of PSD (29.9% and 46.6%, respectively) than susceptible early IV ‘LD00-3309’ (63.9%). These improved breeding lines were derived from PI 587982A. Moreover, early MG V 10049-142-31 (released as DS49-142) had less infection (7.9%) than early V ‘AG5335’ (25.4%), and late MG IV 12060-260-2 (released as DS1260-2) had less infection (19.6%) than susceptible late IV ‘P46T59R’ (55.3%). DS1260–2 was derived from PI 587982A, and DS49-142 was derived from PI 603756. Resistant lines were transferred to breeders for potential use in developing soybean cultivars with resistance to PSD. This research provides useful information for the development of durable resistance to PSD, which will aid in the management of soybean seed decay.

## Introduction

Soybean [*Glycine max (L.)* Merr.] is a major source of plant-based protein and oil for animal and human nutrition, and one of the most economically important oilseed crops grown [1,2]. Worldwide consumption and demand for soybean have markedly increased [3], but high seed quality is crucial for successful soybean production and marketing. Soybean diseases are a threat to both of these, as they can affect not only yield but also seed quality resulting in significant economic losses [https://cropprotectionnetwork.org/yield-loss-calculator/soybean-diseases].

Phomopsis seed decay (PSD) is one of the most damaging soybean seed diseases and causes poor seed quality [4–7]. PSD occurs in almost every soybean growing area worldwide. It is especially prominent in the mid-southern U.S., where warm and humid conditions often occur during late seed fill and full maturity (R8) [8–10]. Rain at this critical time can potentially prevent the entry of harvest machinery into the field and thereby delay harvest. Such conditions favor disease development, causing severe seed decay that negatively impacts soybean production and profit margins [7,11,12]. PSD has caused substantial yield losses to soybean [13–15], although losses have varied across years due to differing environmental conditions. In 2018, soybean yield loss attributed to PSD in the top 29 production states of the U.S was 0.2 million metric tons (MMT) [https://cropprotectionnetwork.org/publications/soybean-disease-loss-estimates-2018], and in 2023, soybean yield losses from PSD were estimated to be about 0.03 MMT [https://cropprotectionnetwork.org/publications/soybean-disease-loss-estimates-from-the-united-states-and-ontario-canada-2023/].

The most characteristic symptoms of PSD include, but are not limited to, seed that is discolored, elongated, shriveled, and cracked. In soybean, seed severely infected with the fungal *Diaporthe*/*Phomopsis* complex can appear moldy with a chalk-white color. However, infected soybean seed may also have no visible symptoms [16]. Soybean seed with symptoms, as well as symptomless seed with infections by the pathogens of the *Diaporthe*/*Phomopsis* complex can both show poor emergence, reduced seedling vigor, damping-off, and seedling death [17]. It has also been reported that oil quality of soybean seed can be reduced, and other seed components negatively altered by infection with *D. longicolla* [18].

The primary causal agent of PSD was first identified as *Phomopsis longicolla* in 1985 [4] but was renamed *Diaporthe longicolla* (Hobbs) J. M. Santos (syn. *Phomopsis longicolla*) in 2011 [19]. Hosseini et al. (2025) used a molecular approach to identify two mating type genes (*MAT1-1-1* and *MAT1-2-1*) in *D. longicolla* isolates and predicted sexual capability of *D. longicolla* [20]. However, information about the sexual structures (such as perithecia) of *D. longicolla* is lacking. Multiple studies have indicated that not only is *D. longicolla* a cause of PSD, but other *Diaporthe* species can also cause PSD, including *D. caulivora* (syn. *D. phaseolorum* var. *caulivora*) and *D. sojae* (syn. *D. phaseolorum* var. *sojae*), as well as other species of *Diaporthe* [7,21–25]. In 1985, Anderson reported that a single seed could be infected by either one pathogen or any combination of *D. caulivora*, *D. sojae*, and *D. longicolla* [26]. Most importantly, research suggests that PSD is more likely the result of a complex interaction of fungal species rather than just a single fungal entity. In studies to evaluate the pathogenicity of *D. longicolla* isolates collected from soybean and non-soybean hosts from different geographic regions, Li et al. (2010, 2018) reported differences among isolates in aggressiveness on soybean [27,28]. In addition, comparison of the mitochondrial genome sequences of eight *D. longicolla* isolates indicated that different numbers of introns and repetitive elements in specific isolates contributed to the differences in genome size [29]. Analysis of whole genome sequences and protein–protein interactions identified conserved global networks and pathogenicity subnetworks in *D. longicolla*, as well as an abundance of genes encoding cell-wall degrading enzymes that play important roles in pathogenicity [30,31].

Common strategies for managing PSD include crop rotation with non-hosts, tillage, fungicide application during pod-fill, and timely harvest shortly after R8 when weather permits [32,33]. Recently, the sensitivities of *D. longicolla* and other *Diaporthe* spp. to difenoconazole and fluopyram fungicides were evaluated, providing valuable information to aid in the development of *Diaporthe* disease management programs [34]. However, control of PSD could also be enhanced by planting PSD-resistant cultivars [35–42]. Multiple exotic soybean accessions [plant introductions (PI)] from the USDA soybean germplasm collection and breeding lines derived from these PIs were screened for more than a decade for seed infection by *Diaporthe* species at Stoneville, Mississippi [11,12,42,43]. The most promising of these are being utilized to develop and release new germplasm lines that have improved adaptation to the mid-southern U.S., along with reduced seed infection caused by *Diaporthe* species. We hypothesized that PSD-resistant gene(s) exist in our improved breeding lines, that can be utilized to develop improved cultivars with resistance to PSD for growers.

To achieve this goal, the specific objectives of this study were (i) to evaluate new improved soybean breeding lines for their reactions to *Diaporthe* spp. infection after inoculation in a 5-year delayed-harvest field trial; and (ii) to identify improved lines with resistance to Phomopsis seed decay. Four soybean lines, DS25−1 (PI 684675), DS31−243 (PI 700941), DS49−142 (PI 703498), and DS1260−2 (PI 705148), tested in this study have been released and are available for research purposes and breeding through the USDA soybean collection via the Germplasm Resources Information Network (GRIN, https://npgsweb.ars-grin.gov/gringlobal/search). These and other breeding lines have been transferred via material transfer agreements to public and private soybean breeders for their potential use in developing soybean cultivars with improved resistance.

## Materials and methods

### Plant materials

A total of 266 soybean entries were evaluated, including breeding lines, cultivars, eight resistant checks, and 12 susceptible checks (S1Table). For comparison, some previously released germplasm lines were also included (S1Table). Available information for these soybean entries, such as accession number, other names, maturity group, and pedigree of entries tested in this study are also in S1Table. As part of an ongoing effort to improve overall seed quality, breeding lines included in this study were selectively advanced based on seed germination data, frequencies of hard seed (impermeable seed coat) and wrinkled seed, as well as on the incidence of Phomopsis seed decay (PSD), in high-temperature environments with natural infection of PSD [43–46]. Impermeable seed coat can delay the uptake of water into the seed and hence delay seed pathogen development, while high temperatures can lower seed viability by directly damaging the seed. Wrinkled seed is evidence of seed damage resulting from weathering and/or heat [45].

Compared to previous studies, the current study used breeding lines derived from additional germplasm sources, as well as material derived from pyramided sources. For example, DS43–72 (11043-225-72) was derived from PIs 417050 and 587982A, 11030-541-28 was derived from PIs 424324B and 587982A, and 16027-312-12 was derived from PIs 594445 and 587982A. Each of these PIs was identified in previous studies and now tied into this study through breeding lines derived from multiple sources. Hence, our current work reports the results of the improved breeding lines derived from these (PIs 587982A, 417050, 424324B, and 594445) and other resistance sources (PI 417479, PI 423941, PI 594619, and PI 603756 [10,39,42]. Further, improved resistant breeding lines were crossed with cultivars and selected for improved seed yield, while also selecting to maintain their improved resistance to PSD. Such was the case for DS1260–2, which was created from DS34–1 × ‘LD00-3309.’ In the pedigree breeding process [47], susceptible breeding lines were dropped when identified. Selected lines received additional testing in yield trials, with some being later shared with public and private soybean breeders. Breeding lines released from our program for improved grain quality include DS25–1, DS31–243, and DS1260–2 (derived from PI 587982A) and DS49–142 (derived from PI 603756) [42]. In addition, the current study covers more years than previous studies. Hence the recommendations for which lines are most useful to breeders are stronger in the current study because they are based on more data.

### Field experiments

Field experiments for evaluation of soybean lines were conducted at Stoneville, Mississippi (MS) on a Sharkey clay soil (very-fine, smectitic, thermictic Chromic Epiaquert) from 2019 to 2023. Planting and harvest dates of the field trials are listed for each year from 2019 to 2023 in Table 1. An Almaco plot planter (model AJ4RP2) with research cones was used for planting. The seeding rate was 25 seed m^-1^ of row. Plots (experimental units) consisted of single rows 3 m long with a row spacing of 0.66 m. A total of 798 plots were tested over the five-year field trial (2019–2023). Furrow irrigation was used to apply water as needed throughout the growing season to alleviate moisture-deficit stress. At the R3 to R5 growth stages [10], inoculum of *D. longicolla,* as described below, was applied to all plots. To promote pathogen infection, irrigation with an overhead watering system commenced after plots were inoculated [42].

**Table 1:** Planting, inoculation, and delayed harvest dates of the field trials from 2019 to 2023.

Tests ^z^	Year	Planting Date	Inoculation date	Delayed harvest date
Early	2019	29-April	24 July	2 Sep – 30 Sep
	2020	5 May	22 July	21 Aug – 2 Oct
	2021	30 April	30 July	31 Aug – 7 Oct
	2022	28 April	3 August	25 Aug – 3 Oct
	2023	19 April	20 July	11 Aug – 28 Sep
				
Late	2019	29 April	24 July	30 Sep – 10 Oct
	2020	5 May	22 July	17 Sep – 11 Oct
	2021	30 April	30 July	2 Sep – 12 Oct
	2022	28 April	3 August	27 Sep – 24 Oct
	2023	19 April	20 July	14 Sep – 20 Oct

^**z**^ Early: tested soybean entries from late maturity group (MG) III through early MG IV; Late: tested soybean entries from late MG IV through early MG V. Each plot was hand-harvested two weeks after the R8 date of the individual plot [10]. The harvest dates reflect the maturity range of the lines screened.

A randomized complete block design (RCBD) with three replications was utilized each year. In 2019 and 2020 the nursery was set up as one trial combining both early (MG III) and late (MGs IV and V) maturing lines. From 2021 through 2023, the nursery was divided into an early maturing trial (Early Test; MG III and early MG IV) and a later maturing trial (Late Test; late MG IV and early MG V). Each plot was hand-harvested two weeks after the R8 (full maturity) date of that plot [10], which was referred to as a “delayed harvest” in this study. Harvested plant materials were placed in an air-conditioned humidity-controlled building. They were stored and allowed to uniformly dry, preparatory to being threshed in a bundle thresher (Almaco Low Profile Plot Thresher). Once threshed, seed was stored at 21°C and 60% relative humidity. They were assayed for Phomopsis seed decay, caused by species in the *Diaporthe/Phomopsis* complex, as described below.

Weather data of total precipitation, number of rainy days, average maximum temperatures, and maximum relative humidity during the growing seasons were obtained from the Stoneville, MS, weather station (https://deltaweather.extension.msstate.edu/stoneville-aws).

### Inoculum preparation and application

The isolate MSPL 10−6 of *D. longicoll*a, one of the most aggressive isolates in our tests [28], was originally collected from field-grown soybean plants at Stoneville, MS and was used for inoculation in the experiments. Inoculum was prepared as previously reported [11,43,44] but is briefly described as follows: isolate was grown at 24°C on potato dextrose agar (Difco Laboratories, Detroit, MI) that was adjusted to pH 4.8 with 25% lactic acid (APDA) after autoclaving. Sporulation of the culture was induced under a fluorescent light output of 300 µmol m^-2^ s^-1^ with a 12-h photoperiod for 30–45 days. Sporulating cultures in each Petri dish were then flooded with approximately 25 ml sterile deionized water, then the fungal colony was gently scraped with a sterile Corning spatula (Corning Inc. Corning, NY) to dislodge conidiospores, which were then filtered with four layers of sterile cheesecloth to eliminate the agar. Concentrations of conidiospores were adjusted to approximately 1.0 × 10^6^/ml in 2019 and 1.0 × 10^5^/ml in 2020–2023, which were determined using a hemocytometer (Hausser Scientific, Blue Bell, PA). A backpack sprayer (Stihl, Model SG 20, Virginia Beach, VA) was used for field inoculations. The sprayer consisted of a hand-held boom containing a single nozzle with an adjustable orifice. In each plot, the conidiospore suspension was sprayed directly onto the soybean pods under the canopy and then evenly across the top of the canopy. Each plot was sprayed using approximately 86 ml of the conidiospore suspension.

### Seed plating assays

Soybean seed samples (25 randomly chosen seeds per plot) that excluded mechanically damaged seed, such as seed with splits or cracked seed coats, were collected from each manually-harvested and threshed plot. Seed plating assays were conducted to determine the percentage of seed infection by *Diaporthe* spp. following a previously reported method [11,12,43,44]. Briefly, seed were surface-disinfected in 0.5% sodium hypochlorite for 3 min, rinsed in sterile distilled water three times, and then placed on APDA [11,12,43,44]. Five seeds were placed uniformly on APDA in each 100 mm × 15 mm Petri dish. Hence, five Petri dishes of five seeds per dish were used to estimate the infection severity of each plot. All seed plates were incubated for four days at 24°C. The number of seeds infected with *Diaporthe* spp. was recorded and the infection rate was expressed as a percentage of seed infection by *Diaporthe* spp. including the inoculated *D. longicolla* isolate and naturally occurring members of the *Diaporthe/Phomopsis* complex in the field. Identification of those PSD-causing pathogens at the *Diaporthe* genus level was accomplished using both cultural morphology and the sequence analysis of the internal transcribed spacer (ITS) regions as previously reported [44]. Briefly, 10 putative *D. longicolla* colonies from the seed assay plates with similar cultural morphology to the MSPL10−6 isolate, along with the type strain TWH P74 and isolate MS-SSC91 of *D*. *aspalathi,* that causes stem canker on soybean, were transferred to PDA or water agar with autoclaved soybean stem pieces or Williams 82 seeds for 45 days under incubation conditions [44]. Observations under the Olympus SZX12 dissecting microscope were conducted from 10 to 20 days after incubation to determine if any isolates formed perithecia. Perithecia have not been found in *D*. *longicolla* but have been reported to be produced by other fungal pathogens in the *Diaporthe-Phomopsis* complex, such as *D*. *aspalathi* and *D*. *caulivora.* Extraction of total genomic DNA from four putative *D*. *longicolla* isolates was carried out as described previously [30,31,44]. Mycelial plugs (3-mm in diameter) from the margins of 10-day old cultures on APDA were cut and placed in potato dextrose broth (Difco Laboratories, Detroit, MI). After 4 days of incubation at 24°C under 12-h light-and dark cycles, mycelia were collected on sterile cheesecloth, washed with sterile water, immediately frozen with liquid nitrogen, and lyophilized with a freeze-drier (IMC Instruments, Inc., Wisconsin, USA). Fungal mycelia were ground with a mortar and pestle and pulverized in liquid nitrogen. Genomic DNA was extracted using a Qiagen DNeasy Plant Mini Kit (Qiagen Inc., Valencia, CA) following the manufacturer’s instruction and qualified with Nanodrop (Thermo Scientific, Waltham, MA, USA) [30,31,44].

### Data analysis

A generalized linear mixed model (GLMM) with binomial error distribution and logit link function was fitted to the combined data of percentage of seed infection by *Diaporthe* spp. from all five years, including all checks, using the package glmmTMB v1.1.11 [48] in R software v4.5.0 [49]. The response variable was the percentage of seed infection by *Diaporthe* spp. out of the total number of seeds in each of the five Petri dishes for each replication of each soybean entry. Entry, year (discrete), test (Early Test vs. Late Test) and their interactions were treated as fixed effects. A random intercept was fitted for each replicate nested within year. The resulting mixed-model equation for the probability of infection for entry *i* in test *j* in year *k* in replicate *l* was:


$$\begin{array}{c} \text{logit }\pi_{ijkl}=\beta_{\text{1},i}+\beta_{\text{2},j}+\beta_{\text{3},k}+\beta_{\text{4},ij}+\beta_{\text{5},ik}+\beta_{\text{6},jk}+\beta_{\text{7},ijk}+u_{kl};\;y_{ijkl}\sim \text{Binomial}(n_{i},\pi_{i}) \end{array}$$

where the indexed β coefficients are the fixed effect terms for the main effects and interactions, and the *u* term is the random intercept.

Because there were some cases of complete separation where some entry-year combinations had zero observed pathogens for all replicates, a weakly informative normal prior distribution with mean 0 and standard deviation 5 was assigned to the fixed effects to regularize the estimates. Simulated residuals were used to test for overdispersion; the estimated dispersion φ = 1.07 was sufficiently close to the value of φ = 1 assumed by the binomial GLMM. Marginal means for each entry were estimated, first individually within the Early Test and the Late Test for each year and then averaged over all Early Tests and Late Tests across all years, inverted from the logit scale. Percentages of seed infection by *Diaporthe* spp. presented in the results represent modeled marginal estimates of infection probability. The z-statistic was used for pairwise mean comparisons and to construct 95% asymptotic confidence intervals around the mean estimates. The Sidak adjustment was used to adjust the 95% confidence intervals for multiple comparisons. Pairwise comparisons for all entries within and across years were taken as odds ratios. Multiple comparison letters were also generated to summarize the significant difference between means, both within each year and averaged across years. Joint chi-squared deviance tests were conducted to assess whether variances due to entry were significant within each test in each year. All post-hoc analyses were done using the R package emmeans v1.11.1 [50].

## Results

From the seed plating assay, 10 putative *D*. *longicolla* colonies that had typical morphology similar to isolate MSPL 10−6 and the type strain TWH P74 (ATCC 60325) were reisolated. None of them produced perithecia from 10 to 45-day old cultures on either PDA plates or water agar with soybean stem pieces as observed under the Olympus SZX12 dissecting microscope. However, *D*. *aspalathi* isolate MS-SSC91, the causal agent of stem canker, formed perithecia in the side-by-side comparison with isolates of *D*. *longicolla*. Results of analyses of DNA sequences of four putative *D*. *longicolla* isolates were identical with our previous *D*. *longicolla* isolates, deposited to Genbank, at the ITS region (Accession MF134860) and in the *TEF-1α* gene (Accession MF189565) [44]. Other unidentified *Diaporthe* spp, with similar morphology were also found. Therefore, even though *D*. *longicolla* was the prevalent species used for inoculation, the data presented in this paper are the percentage of seed infection by *Diaporthe* spp.

The daily maximum air temperatures for the growing season of April through October in 2019 averaged 30.0˚C and ranged from 23.1˚C to 35.7˚C, with similar temperatures observed across the remaining years of the study period. The average daily maximum air temperatures for August 2019–2023 were between 32.1˚C and 35.2 ˚C, much higher than the 24˚C, optimal temperature for PSD. In 2019, totals for precipitation in June, July, and August were 172, 121, and 92 mm, respectively, followed by 161, 67, and 173 mm in 2020; 129, 200, and 142 mm in 2021; 108, 113, and 77 mm in 2022; and 85, 59, and 74 mm in 2023 (S1 Fig.). Maximum relative humidities (RH) in each month across all years were generally in the mid-to-upper 90s%, ranging from 92 to 99%. However, RHs for October of 2022 and 2023 were lower at 88% and 90%, respectively.

Joint deviance tests of seed infection by *Diaporthe* spp. for the tests in each year (2019–2023) indicated that there were significant differences (*P* < 0.05) among entries. The mean percentage of seed infection in each year is shown in Tables 2–6.

**Fig 1:**
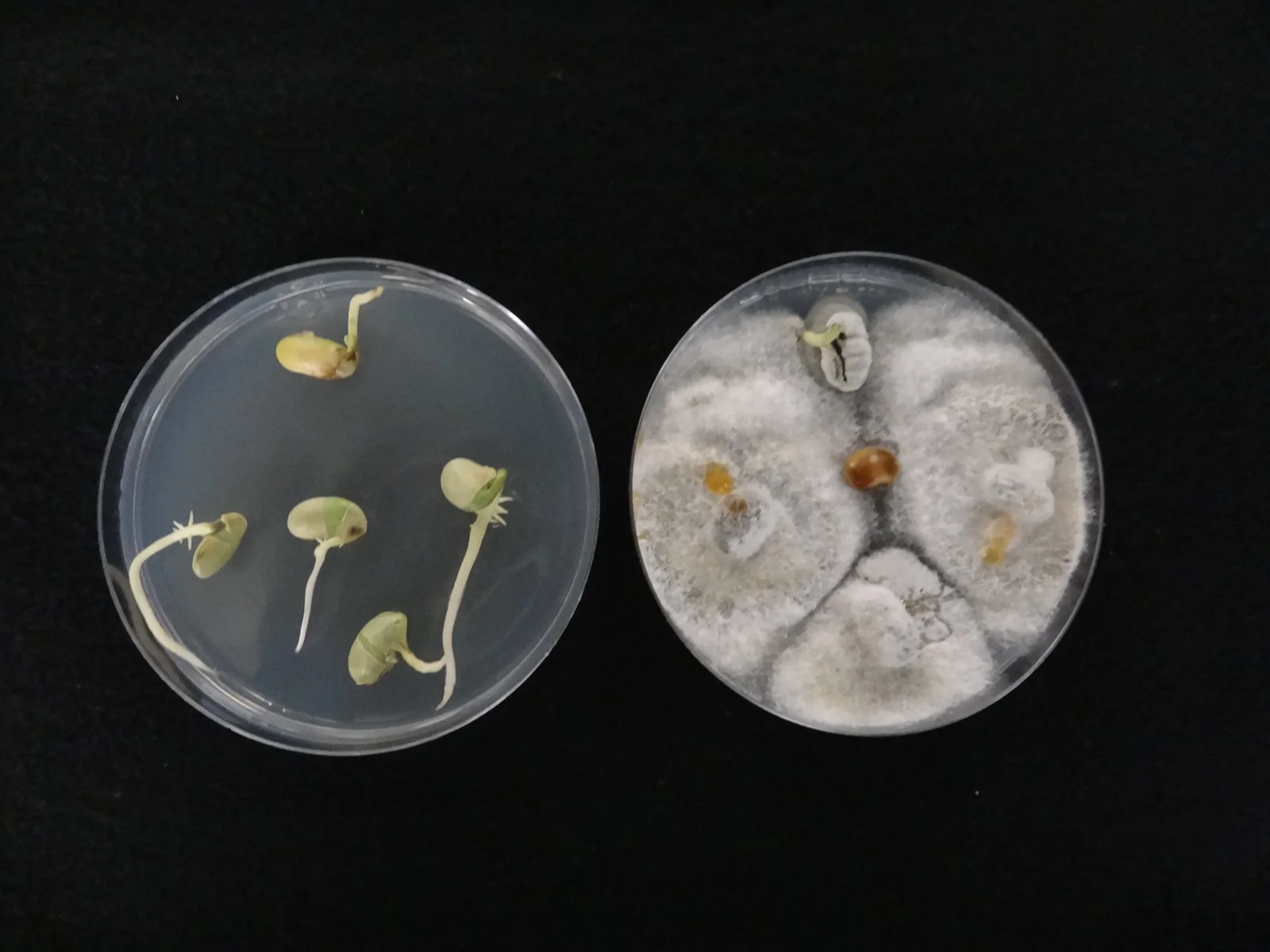
Reaction of resistant vs. susceptible soybean entries to Phomopsis seed decay using the seed plating assay. Resistant line 10049-142-31 (DS49-142) lacked *Diaporthe* spp. (left), while susceptible cultivar AG 5335 manifested the pathogen (right). Seeds were collected from a replicated field test with inoculation treatment and delayed harvest at Stoneville, Mississippi. The photo was taken 7 days after the seed plating assay.

In 2019, the range of seed infections was from 5.4 to 94.4% among entries in the Early Test for soybean MGs III through early IV. The impermeable seed coat-resistant check PI 594619 [51] had the lowest level (5.4%) of seed infection by *Diaporthe* spp., whereas susceptible check ‘LD06-7620’ had the greatest percentage of seed infection (94.4%). Three other susceptible checks, ‘Progeny 4211’, ‘Pella 86’, and ‘CZ3841LL’, had seed infections of 75.8, 81.2, 86.5%, respectively. Among soybean entries from MGs late IV through early V in the Late Test, breeding line DA1239−09 had the lowest score of seed infection (6.7%), which was similar to resistant check 10049-142-31 (8.1%), whereas the susceptible check of comparable maturity, ‘AG5335’, had 29.3% seed infection (Fig 1). The susceptible check ‘P46T59R’ had the greatest score of seed infection (60.0.%). Other susceptible checks, ‘P48A60X’ and ‘AG4632’, had seed infection scores of 55.8 and 58.6%, respectively (Table 2).

**Table 2:** Mean percent seed infection by *Diaporthe* spp. on soybean breeding lines along with resistant and susceptible checks in replicated field tests with inoculation treatment and delayed harvest at Stoneville, Mississippi in 2019.

Entry	Test ^s^	Year	PSD (%) ^t^	SE ^u^	CI lower ^v^	CI upper ^w^	Group ^x^
PI 594619 ^**f**^	Early	2019	5.4	2.6	1.2	21.1	a
DB06x006-93	Early	2019	25.3	5.3	12.8	43.9	ab
10074−112	Early	2019	33.3	5.8	18.7	52.0	bc
10061-236-11	Early	2019	40.0	6.0	23.9	58.6	bcd
11030-541-28	Early	2019	45.3	6.1	28.3	63.5	b-e
10031-243-12	Early	2019	46.5	5.5	31.0	62.7	b-e
13082−1315	Early	2019	52.0	6.2	34.1	69.4	b-f
11043-225-72 ^**y**^	Early	2019	54.7	6.1	36.5	71.7	b-g
65-414-132-1 ^**y**^	Early	2019	55.9	6.1	37.6	72.6	c-g
10061-236-12	Early	2019	55.9	6.1	37.7	72.7	c-g
LD00–3309 ^**z**^	Early	2019	58.2	7.3	36.2	77.3	b-h
SS93–6181 ^**y**^	Early	2019	64.4	5.5	46.8	78.8	d-h
11043-224-91	Early	2019	69.3	5.6	50.6	83.3	d-h
PI 587982A ^**y**^	Early	2019	73.3	5.4	54.7	86.2	e-i
Progeny 4211	Early	2019	75.8	5.2	57.4	87.9	f-i
Pella 86 ^**z**^	Early	2019	81.2	4.7	63.3	91.5	ghi
CZ3841LL ^**z**^	Early	2019	86.5	4.0	69.5	94.8	hi
LG03-4561-14 ^**z**^	Early	2019	94.3	2.6	79.1	98.7	i
LD06–7620 ^**z**^	Early	2019	94.4	2.7	78.9	98.7	i
							
DA1239−09	Late	2019	6.7	2.9	1.6	23.9	a
10049-142-31 ^**y**^	Late	2019	8.1	3.2	2.2	25.1	ab
11030-244-51	Late	2019	9.4	3.4	2.8	26.9	abc
DA13099-008F	Late	2019	9.4	3.4	2.9	26.8	abc
11069-122-11	Late	2019	9.4	3.4	2.9	27.0	abc
11069-323-11	Late	2019	10.8	3.7	3.6	28.6	a-d
12060-260-2	Late	2019	11.6	4.4	3.3	33.9	a-d
11069-333-11	Late	2019	12.1	3.9	4.2	30.1	a-d
12047-1-178	Late	2019	12.1	3.9	4.2	30.1	a-d
12047-1-160	Late	2019	13.4	4.0	4.9	31.6	a-d
11030-242-22	Late	2019	14.6	4.2	5.6	33.2	a-d
DS25−1 ^**y**^	Late	2019	14.8	4.2	5.7	33.2	a-d
11030-242-51	Late	2019	17.3	4.5	7.2	36.2	a-d
DA1221-01-597	Late	2019	18.6	4.7	8.0	37.7	a-d
11030-244-41	Late	2019	22.6	5.1	10.5	42.1	a-d
DA13086-011F	Late	2019	27.9	5.5	14.1	47.7	a-e
AG51X8 ^**z**^	Late	2019	27.9	5.5	14.1	47.7	a-e
DA13086-048F	Late	2019	27.9	5.5	14.1	47.7	a-e
DT97–4290 ^**z**^	Late	2019	28.1	5.5	14.3	47.8	a-e
AG5335 ^**z**^	Late	2019	29.3	5.5	15.1	48.9	a-e
DA1134-015F	Late	2019	30.5	5.6	16.0	50.2	a-e
12060-83-15	Late	2019	30.6	5.6	16.1	50.4	a-e
11069-512-34	Late	2019	31.9	5.7	17.0	51.7	a-e
DA10x30-48F	Late	2019	33.3	5.8	18.0	53.0	a-e
DA10x30-09F	Late	2019	34.4	5.8	18.9	54.2	a-e
11030-244-12	Late	2019	35.9	5.9	20.0	55.7	b-e
DA13062-004F	Late	2019	35.9	5.9	20.0	55.7	b-e
P48A32X	Late	2019	38.6	6.0	22.1	58.2	de
Manokin	Late	2019	40.3	7.2	20.8	63.4	cde
P48A60X ^**z**^	Late	2019	55.8	6.1	36.7	73.4	e
AG4632 ^**z**^	Late	2019	58.6	6.0	39.2	75.7	e
P46T59R ^**z**^	Late	2019	60.0	6.0	40.5	76.7	e

s Early: tested soybean entries from maturity group (MG) III through early MG IV; Late: tested soybean entries from late MG IV through early MG V. Each plot was delay-harvest by hand two weeks after its full maturity date (R8) [10].

t Mean percentage of seed infected by *Diaporthe* spp. from the seed plating assays and analyzed with a generalized linear mixed model (GLMM) with binomial error distribution and logit link function using R software v4.5.0, packages glmmTMB v1.1.11 and emmeans v1.11.1.

u Standard error.

v Lower bound of the 95% confidence interval.

w Upper bound of the 95% confidence interval.

x Same letter indicates no significant difference (*P* < 0.05) after Sidak multiple comparison adjustment.

y Resistant check.

z Susceptible check.

Results from the tests in 2020 are presented in Table 3. Among soybean lines in MGs III through early IV in the Early Test, breeding line 14119-211-10 had the lowest percent seed infection by *Diaporthe* spp. (6.7%). The most susceptible entry in the test was ‘Clark’ maturity isoline L94-1110, which had the greatest percentage of seed infection (65.3%), almost 10 times higher than that of the most resistant breeding line 14119-211-10. In the Late Test for soybean entries of MGs late IV through early V, seed infection by *Diaporthe* spp. ranged from 11.9% (entry 10049-142-31) to 55.9% (entry D13062004F). Resistant check DS25−1 had 22.5% seed infection, which was not different from that of 10049-142-31 (OR = 2.17, 95% CI [0.39, 12.50], *P* = 1.0; Table 3).

**Table 3:** Mean percent seed infection by *Diaporthe* spp. on soybean breeding lines along with resistant and susceptible checks in replicated field tests with inoculation treatment and delayed harvest at Stoneville, Mississippi in 2020.

Entry	Test ^s^	Year	PSD (%) ^t^	SE ^u^	CI lower ^v^	CI upper ^w^	Group ^x^
14119-211-10 ^**y**^	Early	2020	6.7	2.9	1.7	23.3	a
14119-211-24	Early	2020	12.0	3.9	4.3	29.6	ab
10061-236-12	Early	2020	13.4	4.0	5.0	31.2	ab
65-414-132-1 ^**y**^	Early	2020	14.7	4.2	5.8	32.7	abc
D13086011F	Early	2020	17.3	4.5	7.3	35.7	a-d
10074−112	Early	2020	20.0	4.8	9.0	38.7	a-d
D06x006-93	Early	2020	20.0	4.8	9.0	38.7	a-d
SS93–6181 ^**y**^	Early	2020	20.0	4.8	9.0	38.8	a-d
Progeny 4211 ^**z**^	Early	2020	21.4	4.9	9.9	40.3	a-d
D13086048F	Early	2020	24.0	5.2	11.6	43.0	a-e
10031-243-12	Early	2020	24.1	4.9	12.2	42.0	a-e
1130-851-111	Early	2020	28.0	5.5	14.4	47.2	a-e
P40A47X	Early	2020	29.3	5.5	15.4	48.6	a-e
L74-441	Early	2020	31.2	5.5	17.1	50.1	a-e
LD06–7620 ^**z**^	Early	2020	36.1	5.9	20.5	55.4	b-f
L66-432	Early	2020	37.3	5.9	21.4	56.5	b-f
L80-5914	Early	2020	37.4	5.9	21.4	56.6	b-f
CZ3841LL ^**z**^	Early	2020	38.7	6.0	22.5	57.9	b-f
L63-2404	Early	2020	40.0	6.0	23.5	59.1	b-f
Y227-1	Early	2020	42.6	6.1	25.7	61.5	c-f
Pella 86 ^**z**^	Early	2020	42.7	6.1	25.7	61.6	c-f
LG03-4561-14 ^**z**^	Early	2020	42.8	6.1	25.8	61.7	c-f
Clark	Early	2020	46.7	6.1	29.0	65.2	def
L63-3117	Early	2020	53.3	6.1	34.8	71.0	ef
L94-1110	Early	2020	65.3	5.8	46.0	80.6	f
							
10049-142-31 ^**y**^	Late	2020	11.9	3.8	4.1	29.7	a
11069-323-11	Late	2020	13.2	4.0	4.9	31.2	ab
DA15-x085-54	Late	2020	15.9	4.4	6.4	34.4	abc
11069-122-11	Late	2020	17.2	4.5	7.2	35.8	abc
12060-260-2	Late	2020	17.2	4.5	7.2	35.8	abc
12047-1-160	Late	2020	17.2	4.5	7.2	35.8	abc
L74-441	Late	2020	18.1	4.5	8.0	36.0	abc
D13099008F	Late	2020	20.0	4.8	8.9	38.9	a-d
DS25−1 ^**y**^	Late	2020	22.5	5.1	10.6	41.7	a-d
AG4632 ^**z**^	Late	2020	22.6	5.1	10.6	41.8	a-d
14119-721-61	Late	2020	23.9	5.2	11.5	43.2	a-d
P48A60X ^**z**^	Late	2020	25.3	5.3	12.4	44.7	a-e
AG5335 ^**z**^	Late	2020	26.6	5.4	13.3	46.0	a-e
D1488-0228	Late	2020	26.7	5.4	13.4	46.1	a-e
Manokin	Late	2020	27.9	5.5	14.3	47.4	a-e
D1134-015F	Late	2020	27.9	5.5	14.3	47.4	a-e
11069-333-11	Late	2020	30.5	5.6	16.1	50.0	a-e
12047-1-178	Late	2020	31.8	5.7	17.1	51.4	a-e
D1486-10	Late	2020	33.3	5.8	18.2	52.8	a-e
P46T59R ^**z**^	Late	2020	34.6	5.8	19.2	54.1	a-e
D13092-015	Late	2020	37.3	5.9	21.2	56.7	a-e
D13092-039	Late	2020	37.3	5.9	21.2	56.7	a-e
D13062-015	Late	2020	38.6	6.0	22.3	58.0	a-e
L65-3336	Late	2020	39.9	6.0	23.3	59.2	a-e
D13076-042	Late	2020	41.3	6.1	24.4	60.5	b-e
D13062-001	Late	2020	43.9	6.1	26.6	62.9	cde
L92-1195	Late	2020	47.9	6.2	29.9	66.5	de
D13062004F	Late	2020	55.9	6.1	36.9	73.3	e

s Early: tested soybean entries from maturity group (MG) III through early MG IV; Late: tested soybean entries from late MG IV through early MG V. Each plot was delay-harvested by hand two weeks after its full maturity date (R8) [10].

t Mean percentage of seed infected by *Diaporthe* spp. from the seed plating assays and analyzed with a generalized linear mixed model (GLMM) with binomial error distribution and logit link function using R software v4.5.0, packages glmmTMB v1.1.11 and emmeans v1.11.1.

u Standard error.

v Lower bound of the 95% confidence interval.

w Upper bound of the 95% confidence interval.

x Same letter indicates no significant difference (*P* < 0.05) after Sidak multiple comparison adjustment.

y Resistant check.

z Susceptible check.

In 2021, the overall seed infection by *Diaporthe* spp. in the Early Test for soybean entries of MGs III through early IV was more severe than in other years (Table 4). Entry LG03-4561-14 [46] had the greatest seed infection of 98.7%, whereas the lowest entry (15097-225-21) had seed infection of 18.6%. Resistant check SS93−6181 (derived from PI 417479) had 77.5% seed infection in 2021, 64.4% in 2019 (Table 2), 20.0% in 2020 (Table 3), 34.6% in 2022 (Table 5), and 42.8% in 2023 (Table 6), demonstrating how each year’s environment can differ and result in differing severities of the same “resistant” line across environments. Results from the Late Test for soybean entries of MGs late IV through early V indicated that most entries had relatively high levels of seed infection, which obscured meaningful differences among the lines. The range of seed infections was from 34.5% (entry 10049-142-31) to 89.1% (entry AG4632). The resistant check DS25−1 had a seed infection of 66.7%, which was significantly (OR = 3.80, 95% CI [1.04, 13.93], *P* = 0.03) greater than the infection in 10049-142-31, but not significantly (OR = 0.24, 95% CI [0.04, 1.59], *P* = 0.75) different from that of AG4632 (Table 4).

**Table 4:** Mean percent seed infection by *Diaporthe* spp. on soybean breeding lines along with resistant and susceptible checks in replicated field tests with inoculation treatment and delayed harvest at Stoneville, Mississippi in 2021.

Entry	Test ^s^	Year	PSD (%) ^t^	SE ^u^	CI lower ^v^	CI upper ^w^	Group ^x^
15097-225-21	Early	2021	18.6	4.7	8.1	37.4	a
DA15-x085-54	Early	2021	48.3	6.0	30.6	66.5	ab
10074−112	Early	2021	50.7	6.2	32.3	68.9	bc
16028-35-31	Early	2021	53.4	6.2	34.6	71.3	bcd
1419-211-521 ^**y**^	Early	2021	53.4	6.2	34.6	71.3	bcd
14119-211-10	Early	2021	57.4	6.1	38.3	74.6	b-e
1130-528-151	Early	2021	64.1	5.9	44.6	79.9	b-f
15087-511-46	Early	2021	66.8	5.8	47.2	81.9	b-g
65-414-132-1 ^**y**^	Early	2021	69.5	5.6	49.9	83.9	b-g
15087-511-51	Early	2021	72.1	5.5	52.6	85.8	b-g
10031-243-12	Early	2021	72.8	5.1	54.5	85.7	b-g
DA1602–16-B7	Early	2021	74.8	5.3	55.4	87.6	b-g
L74-441	Early	2021	74.8	5.3	55.4	87.7	b-g
SS93–6181 ^**y**^	Early	2021	77.5	5.0	58.3	89.5	b-g
DA1602–13-B7	Early	2021	78.8	4.9	59.7	90.3	c-g
Clark	Early	2021	78.8	4.9	59.8	90.3	c-g
L63-2404	Early	2021	81.5	4.7	62.7	92.0	d-g
Progeny 4211 ^**z**^	Early	2021	81.5	4.7	62.7	92.0	d-g
LD00–3309 ^**z**^	Early	2021	85.5	4.2	67.2	94.4	efg
DA1602–08-B7	Early	2021	86.8	4.0	68.8	95.1	fg
L66-432	Early	2021	88.1	3.8	70.3	95.8	fg
L94-1110	Early	2021	88.1	3.8	70.4	95.9	fg
DA1602–14-B7	Early	2021	89.4	3.6	71.9	96.5	fg
DA1602–15-B7	Early	2021	90.7	3.4	73.5	97.2	fg
L63-3117	Early	2021	90.7	3.4	73.5	97.2	fg
L80-5914	Early	2021	92.1	3.2	75.1	97.8	g
CZ3841LL ^**z**^	Early	2021	93.4	2.9	76.7	98.4	g
LG03-4561-14 ^**z**^	Early	2021	98.7	1.3	77.5	99.9	fg
							
10049-142-31 ^**y**^	Late	2021	34.5	5.8	19.2	53.9	a
DA15-x085-54	Late	2021	41.0	5.9	24.6	59.7	ab
12060-260-2	Late	2021	47.9	6.2	30.0	66.4	abc
DA1585-564F	Late	2021	48.0	6.2	30.1	66.4	abc
DA13099-008F	Late	2021	49.3	6.2	31.2	67.5	abc
AG5335 ^**z**^	Late	2021	52.0	6.2	33.6	69.9	a-d
L65-3366	Late	2021	58.7	6.1	39.6	75.4	a-e
1335-331-422	Late	2021	58.8	6.1	39.7	75.5	a-e
AG55X7	Late	2021	62.8	5.9	43.5	78.7	a-e
DA1541-102F	Late	2021	64.1	5.9	44.8	79.7	a-e
DA13092-015F	Late	2021	65.2	5.8	45.8	80.5	a-e
DS25−1 ^**y**^	Late	2021	66.7	5.8	47.3	81.7	b-e
D1405-212-3F	Late	2021	66.8	5.8	47.4	81.8	b-e
D1405-221-1F	Late	2021	70.8	5.6	51.5	84.7	b-e
DA1568-112F	Late	2021	72.1	5.5	52.8	85.7	cde
DA1134-015F	Late	2021	73.4	5.4	54.2	86.6	cde
11069-323-11	Late	2021	74.7	5.3	55.5	87.5	cde
DA13062-001F	Late	2021	76.1	5.2	57.0	88.4	cde
DA13092-039F	Late	2021	76.1	5.2	57.0	88.4	cde
L92-1195	Late	2021	77.4	5.1	58.5	89.3	cde
DA1539-109F	Late	2021	81.3	4.7	62.7	91.8	de
P46T59R ^**z**^	Late	2021	82.8	4.5	64.4	92.7	e
P48A60X ^**z**^	Late	2021	84.1	4.4	65.9	93.5	e
D1488-0228	Late	2021	85.4	4.2	67.5	94.3	e
AG4632 ^**z**^	Late	2021	89.1	4.3	67.8	97.0	e

s Early: tested soybean entries from maturity group (MG) III through early MG IV; Late: tested soybean entries from late MG IV through early MG V. Each plot was delay-harvested by hand two weeks after its full maturity date (R8) [10].

t Mean percentage of seed infected by *Diaporthe* spp. from the seed plating assays and analyzed with a generalized linear mixed model (GLMM) with binomial error distribution and logit link function using R software v4.5.0, packages glmmTMB v1.1.11 and emmeans v1.11.1.

u Standard error.

v Lower bound of the 95% confidence interval.

w Upper bound of the 95% confidence interval.

x Same letter indicates no significant difference (*P* < 0.05) after Sidak multiple comparison adjustment.

y Resistant check.

z Susceptible check.

**Table 5:** Mean percent seed infection by *Diaporthe* spp. on soybean breeding lines along with resistant and susceptible checks in replicated field tests with inoculation treatment and delayed harvest at Stoneville, Mississippi in 2022.

Entry	Test ^s^	Year	PSD (%) ^t^	SE ^u^	CI lower ^v^	CI upper ^w^	Group ^x^
16031-131-2	Early	2022	1.5	1.4	0.1	21.3	ab
DA1570-25F	Early	2022	1.5	1.4	0.1	21.3	ab
DA1570-29F	Early	2022	2.8	1.9	0.3	19.8	a
DA1571-18F	Early	2022	2.8	1.9	0.3	19.8	a
16031-131-12	Early	2022	4.1	2.3	0.7	20.5	a
DA1571-10F	Early	2022	4.1	2.3	0.7	20.6	a
16016-114-21	Early	2022	4.1	2.3	0.7	20.6	a
DA1572-403F	Early	2022	13.2	4.0	4.9	31.1	abc
1587-513-913	Early	2022	18.5	4.7	8.0	37.2	a-d
16023-617-32	Early	2022	19.8	4.8	8.8	38.6	a-d
1587-513-922	Early	2022	19.8	4.8	8.8	38.7	a-d
DA1579-08F	Early	2022	29.2	5.6	15.2	48.7	a-e
Progeny 4211 ^**y**^	Early	2022	30.6	5.6	16.2	50.1	a-f
SS93–6181	Early	2022	34.6	5.8	19.1	54.1	b-f
L74-441	Early	2022	37.2	6.0	21.2	56.7	c-f
10031-243-12	Early	2022	44.6	5.8	27.8	62.6	d-g
14119-211-10	Early	2022	46.6	6.2	28.8	65.4	d-h
Clark	Early	2022	52.1	6.2	33.5	70.1	e-i
L66-432	Early	2022	53.4	6.2	34.6	71.2	e-j
65-414-132-1	Early	2022	56.1	6.1	37.1	73.4	e-j
L63-3117	Early	2022	58.8	6.1	39.6	75.6	e-j
L94-1110	Early	2022	61.5	6.0	42.1	77.8	f-j
LD00–3309 ^**y**^	Early	2022	69.5	5.6	50.0	83.9	g-j
13099-263-11	Early	2022	76.2	5.2	56.9	88.5	hij
L63-2404	Early	2022	76.2	5.2	57.0	88.6	hij
L80-5914	Early	2022	77.4	5.0	58.4	89.4	ij
LG03-4561-14 ^**y**^	Early	2022	83.0	4.5	64.5	92.9	j
							
D49-2491	Late	2022	0.3	0.6	0.0	55.0	a
DA13092-015F	Late	2022	0.5	0.8	0.0	31.8	a
L65-3366	Late	2022	1.5	1.4	0.1	21.4	a
DA1134-015F	Late	2022	1.5	1.4	0.1	21.3	a
DA10x30-09F	Late	2022	1.5	1.4	0.1	21.2	a
DA1541-102F	Late	2022	1.5	1.4	0.1	21.2	a
13072-293-12	Late	2022	1.5	1.4	0.1	21.2	a
16027-312-12	Late	2022	2.7	1.8	0.3	19.7	a
10049-142-31 ^**z**^	Late	2022	2.7	1.8	0.3	19.7	a
DA13099-008F	Late	2022	2.7	1.8	0.3	19.7	a
DA1488-0228F	Late	2022	2.8	1.9	0.3	19.7	a
Ellis	Late	2022	2.8	1.9	0.3	19.7	a
Hood	Late	2022	2.8	1.9	0.3	19.7	a
DA1539-1010F	Late	2022	2.8	1.9	0.3	19.7	a
DA1539-109F	Late	2022	3.0	1.9	0.4	19.5	a
13072-293-11	Late	2022	4.1	2.3	0.7	20.4	a
DS25−1 ^**z**^	Late	2022	5.3	2.6	1.1	21.6	a
16023-616-42	Late	2022	5.3	2.6	1.1	21.7	a
AG5335 ^**y**^	Late	2022	5.3	2.6	1.1	21.7	a
L92-1195	Late	2022	5.4	2.6	1.1	21.7	a
DA1601−18	Late	2022	6.7	2.9	1.7	23.2	a
P48A60X ^**y**^	Late	2022	9.3	3.4	2.9	26.3	a
AG55X7	Late	2022	9.3	3.4	2.9	26.4	a
12060-260-2	Late	2022	10.5	3.6	3.5	27.8	a
1121-121-111	Late	2022	10.6	3.6	3.5	27.9	a
P46T59R ^**y**^	Late	2022	25.2	5.3	12.4	44.4	a

s Early: tested soybean entries from maturity group (MG) III through early MG IV; Late: tested soybean entries from late MG IV through early MG V. Each plot was delay-harvested by hand two weeks after its full maturity date (R8) [10].

t Mean percentage of seed infected by *Diaporthe* spp. from the seed plating assays and analyzed with a generalized linear mixed model (GLMM) with binomial error distribution and logit link function using R software v4.5.0, packages glmmTMB v1.1.11 and emmeans v1.11.1.

u Standard error.

v Lower bound of the 95% confidence interval.

w Upper bound of the 95% confidence interval.

x Same letter indicates no significant difference (*P* < 0.05) after Sidak multiple comparison adjustment.

y Susceptible check.

z Resistant check.

**Table 6:** Mean percent seed infection by *Diaporthe* spp. on soybean breeding lines along with resistant and susceptible checks in replicated field tests with inoculation treatment and delayed harvest at Stoneville, Mississippi in 2023.

Entry	Test ^s^	Year	PSD (%) ^t^	SE ^u^	CI lower ^v^	CI upper ^w^	Group ^x^
1623-516-141	Early	2023	8.1	3.2	2.3	24.7	a
16023-617-32	Early	2023	8.1	3.2	2.3	24.6	a
1623-516-151	Early	2023	12.0	3.9	4.3	29.4	a
1623-516-143	Early	2023	13.3	4.0	5.0	30.9	ab
65-414-132-1 ^**y**^	Early	2023	13.4	4.0	5.0	31.0	ab
18011-53-41	Early	2023	16.0	4.4	6.6	34.0	abc
18019-45-31	Early	2023	21.3	4.9	9.9	40.0	a-d
11043-225-72 ^**y**^	Early	2023	21.3	4.9	9.9	40.0	a-d
Pella 86 ^**z**^	Early	2023	26.7	5.4	13.6	45.8	a-d
14119-211-10 ^**y**^	Early	2023	27.8	5.4	14.4	46.8	a-d
PI 587982A ^**y**^	Early	2023	32.0	5.7	17.5	51.2	a-d
L66-432	Early	2023	34.7	5.8	19.5	53.7	a-e
LD00–3309	Early	2023	34.7	5.8	19.4	53.8	a-e
18019-45-32	Early	2023	40.0	6.0	23.6	58.9	b-f
18016-41-21	Early	2023	41.3	6.1	24.7	60.1	b-f
SS93–6181	Early	2023	42.8	5.7	26.8	60.5	c-f
DS34−1	Early	2023	44.0	6.1	26.9	62.6	c-f
Clark	Early	2023	45.2	6.1	28.0	63.7	def
L63-2404	Early	2023	49.2	6.1	31.4	67.3	def
18019-45-23	Early	2023	49.3	6.2	31.4	67.4	def
18011-53-21	Early	2023	50.6	6.2	32.5	68.5	def
Progeny 4211 ^**z**^	Early	2023	65.3	5.8	46.2	80.5	ef
LG03-4561-14 ^**z**^	Early	2023	68.1	5.7	48.9	82.6	f
							
10049-142-31 ^**y**^	Late	2023	2.7	1.9	0.3	20.8	ab
16027-312-12	Late	2023	2.7	1.9	0.3	20.4	a
16022-113-23	Late	2023	4.1	2.3	0.7	21.3	a
L65-3366	Late	2023	9.3	3.4	2.8	26.8	abc
16023-616-42	Late	2023	13.2	4.0	4.8	31.6	a-d
16021-234-11	Late	2023	14.7	4.2	5.6	33.3	a-e
DA13099-008F	Late	2023	15.9	4.4	6.3	34.7	a-f
DA1541-102F	Late	2023	21.1	4.9	9.5	40.5	a-g
DA1571-38F	Late	2023	21.3	4.9	9.6	40.7	a-g
12060-260-2	Late	2023	22.5	5.1	10.4	42.1	a-g
L92-1195	Late	2023	22.6	5.0	10.5	42.1	a-g
DS25−1 ^**y**^	Late	2023	25.2	5.3	12.2	44.9	a-h
1587-513-922	Late	2023	26.6	5.4	13.2	46.3	a-h
DA1570-25F	Late	2023	27.7	5.4	14.0	47.5	a-h
DA1601−18	Late	2023	27.8	5.4	14.1	47.6	a-h
DA1579-08F	Late	2023	27.9	5.5	14.1	47.7	a-h
16020-111-22	Late	2023	28.0	5.5	14.1	47.8	a-h
DA1571-10F	Late	2023	30.5	5.6	15.9	50.3	a-h
DA1539-1010F	Late	2023	31.8	5.7	16.9	51.6	a-h
AG5335 ^**z**^	Late	2023	33.3	5.8	18.0	53.1	b-h
L74-441	Late	2023	35.9	5.9	20.0	55.6	c-h
DA1579-04F	Late	2023	37.3	5.9	21.0	57.0	c-i
16016-114-21	Late	2023	39.8	6.0	23.0	59.3	d-i
P48A60X ^**z**^	Late	2023	40.0	6.0	23.1	59.6	d-i
DS-880	Late	2023	44.0	6.1	26.3	63.2	e-i
18019-45-33	Late	2023	44.0	6.1	26.3	63.2	e-i
AG55X7	Late	2023	46.4	6.1	28.4	65.4	f-i
DA1571-16F	Late	2023	46.6	6.1	28.6	65.6	ghi
16018-144-21	Late	2023	55.9	6.1	36.7	73.6	hij
DT97–4290 ^**z**^	Late	2023	69.2	5.6	49.4	83.9	ij
P46T59R ^**z**^	Late	2023	69.3	5.6	49.4	83.9	ij
16019-224-11	Late	2023	81.2	4.7	62.1	92.0	j
17002-14-51	Late	2023	81.2	4.7	62.1	92.0	j

s Early: tested soybean entries from late maturity group (MG) III through early MG IV; Late: tested soybean entries from late MG IV through early MG V. Each plot was delay-harvested by hand two weeks after its full maturity date (R8) [10].

t Mean percentage of seed infected by *Diaporthe* spp. from the seed plating assays and analyzed with a generalized linear mixed model (GLMM) with binomial error distribution and logit link function using R software v4.5.0, packages glmmTMB v1.1.11 and emmeans v1.11.1.

u Standard error.

v Lower bound of the 95% confidence interval.

w Upper bound of the 95% confidence interval.

x Same letter indicates no significant difference (*P* < 0.05) after Sidak multiple comparison adjustment.

y Resistant check.

z Susceptible check.

In 2022, four entries had less than 3% seed infection by *Diaporthe* spp. in the Early Test, 16031-131-2 (1.5%), DA1570-25F (1.5%), DA1570-29F (2.8%) and DA1571-18F (2.8%), whereas the susceptible check of comparable maturity, Progeny 4211, had a seed infection of 30.6% (Table 5). Another susceptible check, LG03-4561-14, had the greatest seed infection of 83.0%. Results of the Late Test for soybean entries of MGs late IV through early V showed lower seed infection compared to tests in other years with a range from 0.3 (D49-2491) to 25.2% (P46T59R), indicating an overall environment of less severity for the late test in 2022. As an example of the effect of environment, the seed infection of susceptible check P46T59R was 25.2% in 2022, but was 60.0% in 2019, 34.2% in 2020, 82.8% in 2021, and 69.3% in 2023 (Tables 2–6). September 2022 had only 1 mm of precipitation and October 2022 had the lowest percent RH (88%) of any single month of the 5-year period (S1 Fig.), possibly contributing to lower levels of seed infection during those periods.

In 2023, the percentage of seed infection by *Diaporthe* spp. ranged from 8.1 to 68.1% in the Early Test, and from 2.7 to 81.2% in the Late Test (Table 6). The three entries with the lowest seed infections in the Early Test were 1623-516-141 (8.05%), 16023-617-32 (8.1%), and 1623-516-151 (12.0%), which all have the hard seed trait (impermeable seed coat) [51] derived from PI 594619 (Table 6). Entries 10049-142-31, released as DS49−142 (resistant check), 16027-312-12, and 16022-113-23 had the lowest seed infection scores of 2.7, 2.7, and 4.1%, respectively, in the Late Test (Table 6). Breeding line 16027-312-12 was derived from two sources of resistance, PI 587982A and PI 594445 (S1 Table), which indicates the potential for stacking multiple sources of resistance in a single line.

**Fig 2:**
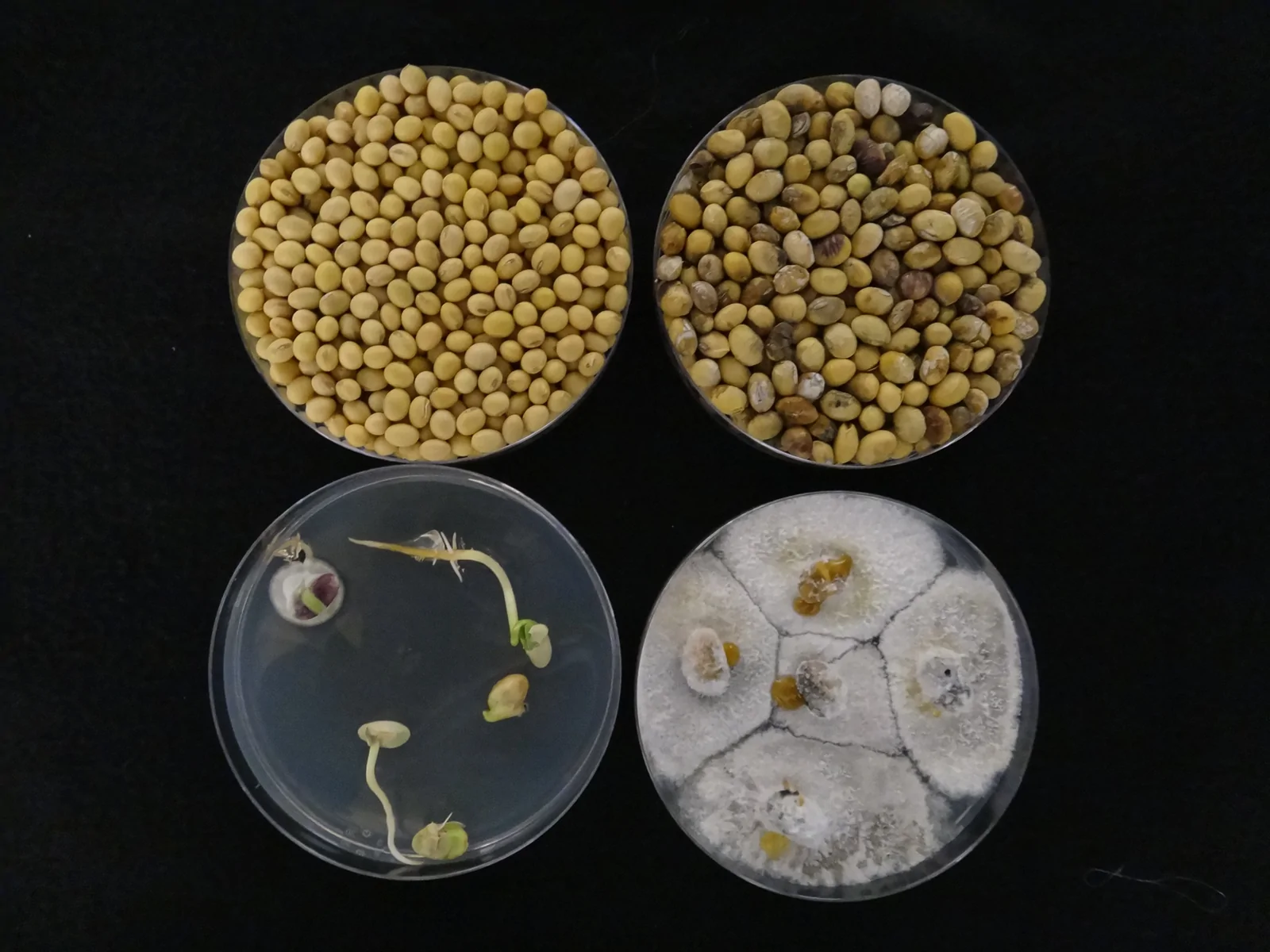
Healthy vs. Phomopsis decayed soybean seeds. Resistant line 12060-260-2 (DS1260−2) (left), and susceptible cultivar P46T59R (right). Seeds were collected from a replicated field test with inoculation treatment and delayed harvest at Stoneville, Mississippi. The photo was taken 7 days after the seed plating assay.

In view of the results of the multi-year average of mean percent seed infection by *Diaporthe* spp. on soybean breeding lines from MGs III through early IV in the Early Test, breeding lines DA1570-25F, DA1571-10F, and 16016-114-21 had less than 5% seed infection across two years (2022 and 2023) (Table 7). In the Late Test, the resistant check 10049-142-31(released as DS49–142, Fig 1), tested in all 5 years, had an overall mean of 7.9%, which was significantly (OR = 0.25, 95% CI [0.08, 0.83], *P* = 0.002) lower than susceptible check AG5335 (25.4%, Fig 1) in five years of testing. Also tested over all five years, entry 12060-260-2 (released as DS1260–2, Fig 2) [42] had significantly (OR = 0.20, 95% CI [0.09, 0.45], *P* < 1 × 10^-13^) lower seed infection by *Diaporthe* spp. pathogens (19.6%) than susceptible check P46T59R (55.3%), which was also tested for five years (Table 7, Fig 2). The modeled probability of soybean percent seed infection by *Diaporthe* spp. in the tests of soybean entries from MGs III through early IV, and from late MGs IV through early V are presented in S2 Fig and S3 Fig, respectively.

**Table 7:** Overall mean percent seed infection by *Diaporthe* spp. on soybean breeding lines along with resistant and susceptible checks in replicated field tests with inoculation treatment and delayed harvest at Stoneville, Mississippi across years in 2019, 2020, 2021, 2022, and 2023.

Entry	Test ^s^	PSD (%) ^t^	SE ^u^	CI lower ^v^	CI upper ^w^	Group ^x^	Years tested
DA1570-25F	Early	1.8	1.5	0.1	23.9	a-e	2022, 2023
DA1571-18F	Early	2.8	1.9	0.3	22.9	ab	2022
DA1570-29F	Early	2.8	1.9	0.3	22.9	ab	2022
16031-131-12	Early	4.1	2.3	0.6	23.2	ab	2022
DA1571-10F	Early	4.3	2.3	0.7	23.1	a	2022, 2023
16016-114-21	Early	4.3	2.3	0.7	23.1	a	2022, 2023
PI 594619 ^y^	Early	5.4	2.6	1.0	24.5	a	2019
1623-516-141	Early	8.1	3.2	2.0	27.3	a-d	2023
1623-516-151	Early	12.0	3.9	3.9	31.8	a-f	2023
14119-211-24 ^y^	Early	12.0	3.9	3.8	31.8	a-f	2020
16023-617-32	Early	12.8	2.9	5.7	26.3	abd	2022, 2023
DA1572-403F	Early	13.2	4.0	4.5	33.1	a-f	2022
1623-516-143	Early	13.4	4.0	4.5	33.3	a-f	2023
18011-53-41	Early	16.0	4.4	6.0	36.4	a-g	2023
D13086011F	Early	17.3	4.5	6.7	37.9	a-i	2020
1587-513-913	Early	18.5	4.7	7.4	39.1	a-k	2022
15097-225-21	Early	18.6	4.7	7.5	39.3	a-h, j, l	2021
1587-513-922	Early	19.9	4.8	8.3	40.7	a-n	2022, 2023
D06x006-93	Early	20.0	4.8	8.3	40.8	a-m	2020
18019-45-31	Early	21.3	4.9	9.1	42.2	a-o	2023
D13086048F	Early	24.0	5.2	10.8	45.1	a-o	2020
DB06x006-93	Early	25.3	5.3	11.7	46.4	a-p	2019
1130-851-111	Early	28.0	5.5	13.5	49.2	a-r	2020
DA1579-08F	Early	29.2	5.5	14.3	50.5	a-s	2022, 2023
P40A47X	Early	29.3	5.5	14.4	50.5	a-s	2020
14119-211-10	Early	29.9	3.4	19.8	42.3	a-o	2020, 2021, 2022, 2023
10061-236-12	Early	30.7	4.6	17.7	47.7	a-q	2019, 2020
10074−112	Early	33.5	3.5	22.9	46.0	a-o	2019, 2020, 2021
11043-225-72 ^y^	Early	36.4	4.5	23.0	52.2	b-s	2019, 2023
65-414-132-1 ^y^	Early	38.6	3.1	28.8	49.4	e-s	2019, 2020, 2021, 2022, 2023
18019-45-32	Early	40.0	6.0	22.2	60.8	c, e-u	2023
10061-236-11	Early	40.0	6.0	22.2	60.8	c-u	2019
18016-41-21	Early	41.3	6.1	23.3	62.0	e-v	2023
Y227-1	Early	42.6	6.1	24.3	63.2	e-w	2020
DS34−1	Early	44.0	6.1	25.4	64.4	f-x	2023
11030-541-28	Early	45.3	6.1	26.5	65.6	f-x	2019
10031-243-12	Early	46.6	3.2	36.2	57.3	j-t	2019, 2020, 2021, 2022
SS93–6181 ^y^	Early	47.7	3.0	37.7	57.9	l-u	2019, 2020, 2021, 2022, 2023
L74-441	Early	48.5	3.8	36.2	61.0	j-u	2020, 2021, 2022, 2023
18019-45-23	Early	49.3	6.2	29.8	69.0	g-x	2023
DA15-x085-54	Early	49.4	6.2	29.8	69.2	g-x	2020, 2021
18011-53-21	Early	50.6	6.2	30.9	70.2	h-y	2023
Pella 86 ^z^	Early	51.3	4.0	38.1	64.4	n-x	2019, 2020, 2023
13082−1315	Early	52.0	6.2	32.0	71.3	h-z	2019
PI 587982A ^y^	Early	53.2	4.7	37.5	68.3	o-x	2019, 2023
1419-211-521	Early	53.4	6.2	33.2	72.5	i, k, m-z	2021
16028-35-31	Early	53.4	6.2	33.2	72.5	i, k, m-z	2021
Progeny 4211 ^z^	Early	55.7	3.1	45.1	65.8	r-x	2019, 2020, 2021, 2022, 2023
L66-432	Early	56.1	3.5	44.1	67.4	p-x	2020, 2021, 2022, 2023
Clark	Early	56.7	3.2	45.7	67.0	r-x	2020, 2021, 2022, 2023
L63-2404	Early	63.5	3.2	52.3	73.4	t-α	2020, 2021, 2022, 2023
LD00–3309 ^z^	Early	63.9	3.4	52.0	74.4	t-α	2019, 2021, 2022, 2023
1130-528-151	Early	64.1	5.9	43.0	80.9	q-β	2021
15087-511-46	Early	66.8	5.8	45.6	82.9	s-β	2021
11043-224-91	Early	69.3	5.6	48.1	84.7	t-β	2019
L63-3117	Early	71.6	3.6	57.9	82.1	v-β	2020, 2021, 2022
15087-511-51	Early	72.1	5.5	50.9	86.6	t-β	2021
L94-1110	Early	73.8	3.3	61.2	83.4	x-β	2020, 2021, 2022
L80-5914	Early	74.2	3.7	60.0	84.7	x-β	2020, 2021, 2022
DA1602–16-B7	Early	74.8	5.3	53.7	88.4	t-β	2021
LD06–7620	Early	75.6	5.2	54.4	88.9	t-β	2019, 2020
13099-263-11	Early	76.2	5.2	55.1	89.3	u-β	2022
DA1602–13-B7	Early	78.8	4.9	57.9	90.9	w-β	2021
CZ3841LL ^z^	Early	79.5	3.5	65.4	88.8	y-β	2019, 2020, 2021
LG03-4561-14	Early	86.2	2.9	73.4	93.4	β	2019, 2020, 2021, 2022, 2023
DA1602–08-B7	Early	86.8	4.0	66.8	95.5	zαβ	2021
DA1602–14-B7	Early	89.4	3.6	69.9	96.8	αβ	2021
DA1602–15-B7	Early	90.7	3.4	71.4	97.4	αβ	2021
							
D49-2491	Late	0.3	0.5	0.0	75.2	a-w	2022
13072-293-12	Late	1.5	1.4	0.1	27.1	a-m	2022
16027-312-12	Late	2.7	1.3	0.5	13.4	a	2022, 2023
DA1488-0228F	Late	2.7	1.9	0.3	23.7	a-g	2022
Ellis	Late	2.7	1.9	0.3	23.7	a-g	2022
Hood	Late	2.7	1.9	0.3	23.7	a-g	2022
13072-293-11	Late	4.0	2.3	0.6	23.8	a-f	2022
16022-113-23	Late	4.1	2.3	0.6	24.1	a-e	2023
DA1239−09	Late	6.7	2.9	1.4	26.3	a-g	2019
10049-142-31 ^y^	Late	7.9	1.7	3.7	16.3	ab	2019, 2020, 2021, 2022, 2023
DA10x30-09F	Late	8.2	3.6	1.7	31.9	a-l	2019, 2022
16023-616-42	Late	8.5	2.4	3.1	21.3	abc	2022, 2023
DA13092-015F	Late	8.6	6.0	0.7	56.8	a-v	2021, 2022
11030-244-51	Late	9.4	3.4	2.5	29.2	a-j	2019
DA1539-1010F	Late	10.4	3.5	3.1	29.2	a-h	2022, 2023
1121-121-111	Late	10.6	3.6	3.1	30.5	a-j	2022
L65-3366	Late	11.4	3.6	3.7	30.2	a-j	2021, 2022, 2023
11069-122-11	Late	12.8	2.9	5.8	26.2	a-f	2019, 2020
DA13099-008F	Late	13.1	2.6	6.5	24.9	a-d	2019, 2021, 2022, 2023
DA1601−18	Late	14.2	3.3	6.2	29.5	a-h	2022, 2023
16021-234-11	Late	14.6	4.2	5.1	35.3	a-l	2023
11030-242-22	Late	14.6	4.2	5.1	35.3	a-l	2019
12047-1-160	Late	15.2	3.0	7.4	28.7	a-g, j	2019, 2020
DA1541-102F	Late	16.2	4.6	5.7	38.1	a-m	2021, 2022, 2023
11030-242-51	Late	17.3	4.5	6.6	38.2	a-m	2019
DA1221-01-597	Late	18.6	4.7	7.4	39.7	a-o	2019
12060-260-2	Late	19.6	2.4	12.6	29.2	a-j	2019, 2020, 2021, 2022, 2023
11069-333-11	Late	19.8	3.6	10.2	34.7	a-l	2019, 2020
D13099008F	Late	20.0	4.8	8.2	41.2	a-m, o	2020
12047-1-178	Late	20.2	3.6	10.5	35.4	a-l	2019, 2020
DA1134-015F	Late	20.8	5.6	7.6	45.6	a-q	2019, 2021, 2022
DA1571-38F	Late	21.3	4.9	9.0	42.6	a-p	2023
DS25−1 ^y^	Late	22.2	2.7	14.3	32.8	b-k	2019, 2020, 2021, 2022, 2023
11030-244-41	Late	22.6	5.1	9.8	44.0	a-p	2019
14119-721-61	Late	23.9	5.2	10.6	45.4	a-p	2020
AG5335 ^z^	Late	25.4	2.8	17.0	36.1	c-m	2019, 2020, 2021, 2022, 2023
L74-441	Late	25.5	3.6	15.0	39.8	c-m	2020, 2021, 2022, 2023
DA15-x085-54	Late	26.2	4.0	14.9	41.9	c-o	2020, 2021
1587-513-922	Late	26.5	5.4	12.3	48.1	b-r	2022, 2023
DA1539-109F	Late	26.6	7.2	9.2	56.3	a-t	2021, 2022
11069-323-11	Late	27.5	3.9	16.2	42.7	c-p	2019, 2020, 2021
DA1570-25F	Late	27.7	5.4	13.1	49.3	b-r	2022, 2023
DA1579-08F	Late	27.9	5.5	13.2	49.5	b-r	2022, 2023
DA13086-048F	Late	27.9	5.5	13.3	49.5	b-r	2019
AG51X8 ^z^	Late	27.9	5.5	13.3	49.5	b-r	2019
DA13086-011F	Late	27.9	5.5	13.3	49.5	b-r	2019
16020-111-22	Late	27.9	5.5	13.3	49.5	b-r	2023
D1134-015F	Late	27.9	5.5	13.3	49.5	b-r	2020
DA1571-10F	Late	30.4	5.6	15.0	52.1	c-s	2022, 2023
12060-83-15	Late	30.6	5.6	15.1	52.2	c-s	2019
11069-512-34	Late	31.9	5.7	16.0	53.5	c-s	2019
L92-1195	Late	32.3	3.8	20.7	46.6	d-q	2020, 2021, 2022, 2023
D1486-10	Late	33.3	5.8	17.0	54.8	c-t	2020
DA10x30-48F	Late	33.3	5.8	17.0	54.8	c-t	2019
Manokin	Late	33.8	4.5	20.4	50.6	d-s	2019, 2020
AG55X7	Late	34.7	4.1	22.3	49.6	e-s	2021, 2022, 2023
11030-244-12	Late	35.9	5.9	18.9	57.4	d-u	2019
DA13062-004F	Late	35.9	5.9	18.9	57.4	d-u	2019
D13092-015	Late	37.3	5.9	19.9	58.6	d-u	2020
D13092-039	Late	37.3	5.9	19.9	58.6	d-u	2020
DA1579-04F	Late	37.3	5.9	19.9	58.6	d-u	2023
D13062-015	Late	38.6	6.0	20.9	59.9	d-v	2020
P48A32X	Late	38.6	6.0	20.9	59.9	d-v	2019
16016-114-21	Late	39.8	6.0	21.8	61.0	f-v	2022, 2023
L65-3336	Late	39.9	6.0	21.9	61.1	e-v	2020
P48A60X ^z^	Late	40.8	3.3	30.1	52.5	l-t	2019, 2020, 2021, 2022, 2023
D13076-042	Late	41.2	6.0	23.0	62.3	g-v	2020
D13062-001	Late	43.9	6.1	25.1	64.7	h-w	2020
DS-880	Late	43.9	6.1	25.1	64.7	i-w	2023
18019-45-33	Late	43.9	6.1	25.1	64.7	i-v	2023
DA1571-16F	Late	46.6	6.1	27.2	67.0	k-w	2023
DA1585-564F	Late	48.0	6.2	28.3	68.3	k-w	2021
DT97–4290	Late	48.4	4.7	32.9	64.2	m-v	2019, 2023
P46T59R ^z^	Late	55.3	3.0	44.8	65.4	r-w	2019, 2020, 2021, 2022, 2023
D13062004F	Late	55.9	6.1	35.1	74.8	n-w	2020
16018-144-21	Late	55.9	6.1	35.1	74.8	n-w	2023
1335-331-422	Late	58.7	6.1	37.6	77.0	p-w	2021
D1488-0228	Late	59.3	5.2	40.9	75.4	q-w	2020, 2021
AG4632 ^z^	Late	60.1	4.7	43.5	74.6	r-w	2019, 2020, 2021
D1405-212-3F	Late	66.8	5.8	45.1	83.0	s-w	2021
D1405-221-1F	Late	70.8	5.5	49.1	85.9	t-w	2021
DA1568-112F	Late	72.1	5.5	50.5	86.8	uvw	2021
DA13062-001F	Late	76.1	5.2	54.6	89.4	vw	2021
DA13092-039F	Late	76.1	5.2	54.6	89.4	vw	2021
16019-224-11	Late	81.2	4.7	60.1	92.5	w	2023
17002-14-51	Late	81.2	4.7	60.1	92.5	w	2023

s Early: tested soybean entries from late maturity group (MG) III through early MG IV; Late: tested soybean entries from late MG IV through early MG V. Each plot was delay-harvested by hand two weeks after its full maturity date (R8) [10].

t Mean percentage of seed infected by *Diaporthe* spp. from the seed plating assays and analyzed with a generalized linear mixed model (GLMM) with binomial error distribution and logit link function using R software v4.5.0, packages glmmTMB v1.1.11 and emmeans v1.11.1.

u Standard error.

v Lower bound of the 95% confidence interval.

w Upper bound of the 95% confidence interval.

x Same letter indicates no significant difference (*P* < 0.05) after Sidak multiple comparison adjustment.

y Resistant check.

z Susceptible check.

## Discussion

Diseases caused by pathogens are the major cause of yield loss and poor seed quality, greatly impairing the stability of crop production in the world. Breeding for plant disease resistance is crucial for sustainable agriculture. Utilizing and releasing disease-resistant lines and cultivars can provide long-term plant protection against pathogens and is a cost-effective and environmentally friendly way to manage crop diseases.

Seed quality is important for successful crop production, which can be affected by plant pathogens, environmental conditions, and their interactions [52,53]. Soybean seed quality has become a growing concern, as poor grain quality can be price-discounted when sold and can also limit the use of harvested seed for replanting [54,55]. Poor soybean quality is often associated with Phomopsis seed decay (PSD). As our specific goal has been to develop and release soybean lines with improved levels of PSD-resistance, we developed protocols to evaluate breeding lines and identify promising lines under field conditions using a multiyear approach.

When field trials are used to identify potentially resistant breeding lines, there are multiple issues to manage. For example, without artificial inoculation, susceptible plants may not always have a similar opportunity to be infected by the pathogen. This may be due to environments that are unfavorable to fungal growth and disease development in certain years. In addition, the pathogen may not be distributed evenly in the field, permitting disease or pathogen escapes and resulting in false identification of host resistance [12,43]. These potential problems need to be managed and identified when they occur. Based on our past years of experience in screening soybean for resistance to PSD in the field at Stoneville, MS [11,12,43,44], we determined that artificial inoculation with the pathogen helps to ensure a more uniform distribution of the pathogen and to reduce the possibility of disease “escapes” [56,57]. Hence, in this study soybean lines were evaluated under inoculated treatments, which increased confidence in the significant differences observed for percentages of seed infection by *Diaporthe* spp. Because different fungal pathogens with similar cultural morphology in the *Diaporthe*/*Phomopsis* complex have recently been reported to cause PSD [24], this study reports the percentage of seed infection by *Diaporthe* spp., as opposed to reporting infection at the species level.

To better manage environmental biases, we inoculated each plot in our field study and harvested each plot two weeks after it matured (R8) [10]. Our previous study [44] indicated that mean seed infection by *D. longicolla* was more severe after delayed harvest than when plots were harvested promptly after maturity. Using delayed harvesting helps ensure more disease severity and reduces the likelihood of “disease escapes,” because moisture close to the seed (due to high humidity or precipitation) after maturity and dry down is a key factor affecting the extent of damage by PSD [58]. In addition, early (April) planting in the midsouth also encourages more seed damage at senescence and maturity because April-planted early-maturing soybean generally mature in August through early September, when the environment is usually hot and humid and can thus be more conducive to severe PSD [59]. However, even when utilizing inoculated plots, overhead irrigation, delayed harvest, and early plantings, the effect of each year’s environment can still promote too little or too much severity such that the statistical separation of “resistant” and “susceptible” genotypes may still be difficult or questionable. Therefore, experimental testing over multiple years (four or five are preferable) is required to separate with confidence resistant and susceptible breeding lines. Many breeding lines can appear favorable after one or two years of testing, but lines that perform well over four or five years of testing may be considered for release and recommended for use. Early IV breeding line 14119-211-10 had a significantly lower percentage of seed infection score (29.9%) over four years of testing than susceptible early IV cultivars Progeny 4211 (55.7% over five years), Clark (56.7% over four years), and LD00−3309 (63.9% over four years of testing) (Table 7). Likewise, late III 65-414-132-1 (DS65−1) [39] and early IV 10031-243-12 (released as DS31−243) [43] had significantly less infection (38.6% and 46.6%, respectively) over five years and four years, respectively, than susceptible late III LG03-4561-14 (86.2% in five years of testing) (Table 7). These three improved breeding lines, all derived from PI 587982A, are recommended for use in developing early-maturing PSD-resistant cultivars.

Later-maturing breeding lines may avoid PSD infection because they senesce in a cooler environment, where *Diaporthe* spp. are generally less active. Even so, our protocol was still able to detect significant differences among late IV and early V soybean lines over multiple years. Over five years of testing, early V 10049-142-31 (released as DS49–142) [42] had a lower percentage of infection (7.9%) than early V cultivar AG5335 (25.4%) (Table 7). Likewise, late IV 12060-260-2 (released as DS1260–2, Fig 2) [42] had a lower percentage of seed infection (19.6%) than susceptible late IVs P48A60X (40.8%) and P46T59R (55.3%) (Table 7). We therefore recommend, based on five years of testing, DS1260–2, derived from PI 587982A, and DS49–142, derived from PI 603756, for use in developing group IV and V soybeans with resistance to PSD.

As shown from limited testing (2022 and 2023) in this work, the hard seed trait (impermeable seed coat) may have potential for preserving seed quality under adverse weather conditions in MG IV soybean in the mid-southern U.S. Potts et al. [60] compared MG V cultivar Dare with hard seeded D67-5677-1 and suggested the possibility of using the hard seed trait for preserving seed quality for soybean in the south. Hartwig and Potts [61] went on to demonstrate that the hard seed trait derived from wild soybean (*Glycine soja* Sieb. and Zucc.) could protect soybean seed viability in soybean lines derived from MG V cultivar Forrest. They found that the hard seed line D81-9776 had 96% viable seed when harvested at maturity and 86% viable seed when harvested four to six weeks after maturity, compared to Forrest, which had 96% and 5% viable seed, respectively. Kebede et al. [51] later identified a single gene in *G. max* PI 594619 that codes for the hard seed trait. The current work tested five improved MG IV hard seeded breeding lines derived from PI 594619. 16023-617-32 (Early Test) and 16023-616-42 (Late Test) were tested across 2022 and 2023 and had PSD infections of 12.8% and 8.5%, respectively, whereas 1623-516-141, 1623-516-151, and 1623-516-143 (all Early Test) were tested only in 2023 and had infections of 8.1%, 12.0%, and 13.4%, respectively. The hard seed trait could be effective in maintaining seed quality under environmental weathering with PSD, but the main impractical issue for using the hard seed trait would be in producing seed beans that would promptly germinate the next year in grower fields. Either mechanical scarification or some sort of seed treatment might likely be needed for prompt and uniform germination after sewing. Under natural conditions in the soil, the hard seed trait breaks down gradually and unevenly, producing gradual and uneven germination that could result in uneven maturation of the crop. Uneven maturation could cause delays in harvest unless a desiccant were applied for uniform crop dry down, which is actually a common current practice in the mid-southern U.S.

To assist in the development of PSD-resistant cultivars, and to aid in studies related to seed quality, improved germplasms late MG IV DS25−1 (PI 684675), early MG IV DS31−243 (PI 700941,10031-243-12), early MG V DS49−142 (PI 703498, 10049-142-31), and late MG IV DS1260−2 (PI 705148, 12060-260-2) were publicly released by USDA and are available for research and breeding purposes from the Germplasm Resources Information Network (GRIN, https://npgsweb.ars-grin.gov/gringlobal/search). Prior to release, these lines were also transferred by Material Transfer Agreement (MTA) to public and private breeding and research entities, both in the USA and internationally. In addition, DS65−1 (65-414-132-1), DS43−72 (11043-225-72), DS34−1 (4034-312-41), 11069-323-11, 11069-333-11, 11069-122-11, 11043-224-91, and 10061-236-11 were transferred by MTA to public and private breeding and research entities for potential use in developing improved cultivars and for ongoing research on seed quality issues. Further, records from the USDA soybean germplasm collection (personal communication from Todd Bedford) indicate that released germplasm lines DS25−1 (PI 684675), DS31−243 (PI 700941), DS49−142 (PI 703498), and DS1260−2 (PI 705148) have all been requested by and transferred to public researchers/breeders. Additionally, DS25−1 and DS1260−2 were requested by and transferred to multiple private researchers/breeders. The transfer of improved material with resistance to PSD to commercial and public breeders with access to the appropriate intellectual property rights for marketing the genetically modified organism (GMO) traits that producers desire represents an important step in the process of developing soybean cultivars for producers with improved resistance to PSD.

In conclusion, developing, releasing and utilizing PSD-resistant cultivars is an economical and environmentally friendly strategy to protect soybean from disease-induced seed damage, especially when using the popular early soybean production system (ESPS) [62] in mid-southern states of the U.S. Because Phomopsis seed decay is one of the most economically important soybean seed diseases in the southern states of the U.S, utilizing effective methods to identify sources of resistance to the fungal pathogens in the *Diaporthe/Phomopsis* complex that causes PSD is essential for the development of durable resistance to PSD and to aid in the management of soybean seed decay during soybean production in the mid-southern U.S.

## Supporting information

S1 Table A list of soybean breeding lines evaluated in this study. Entry: Name of seed samples assayed each year; MG: Maturity group; Early: tested soybean entries from maturity group (MG) III through early MG IV; Late: tested soybean entries from late MG IV through early MG V. Check: S is susceptible check; R is resistant check.
(PDF)

S1 Fig Average daily maximum temperatures, average daily maximum relative humidity, total precipitation and number of rainy days during the soybean growing seasons for the months of April through October from 2019 to 2023 at Stoneville, Mississippi. (DOCX)

S2 Fig The modeled probability of soybean percentage of seed infection by *Diaporthe* spp. in the tests of soybean entries from maturity group (MG) III through early MG IV. Solid error bars represent 95% confidence intervals of each mean, and gray shaded error bars represent 95% comparison intervals: two entry means whose comparison intervals do not overlap are significantly different at the adjusted *P* < 0.05.
(PNG)

S3 Fig The modeled probability of soybean percentage of seed infection by *Diaporthe* spp. in the tests of soybean entries from late maturity group (MG) IV through early MG V. Solid error bars represent 95% confidence intervals of each mean, and gray shaded error bars represent 95% comparison intervals: two entry means whose comparison intervals do not overlap are significantly different at the adjusted *P* < 0.05.
(PNG)
