# Correction: Multi-year evaluation and release of improved soybean lines for resistance to Phomopsis seed decay

**DOI:** 10.1371/journal.pone.0360730

**Published:** 2026-10-13

**Authors:** Shuxian Li, James R. Smith, Anne M. Gillen, Quentin D. Read

The hyperlink in the Data Availability statement is incorrect. The correct hyperlink is: https://doi.org/10.15482/USDA.ADC/32091706.v2.
