# Supplementary material for: Multi-year evaluation and release of improved soybean lines for resistance to Phomopsis seed decay
Source: PLoS One. 2026 Aug 24;21(8):e0354166. doi: 10.1371/journal.pone.0354166 (PMC13502683; doi:10.1371/journal.pone.0354166)
Supplement: Supplementary file 1 [file pone.0354166.s001.pdf]

S1 Table. A list of soybean entries evaluated in this study

		Year	Entry*	Accession/other names	MG**	Check**	Test***	Pedigree	Note
1	1	2019	10031-243-12	DS31-243 (PI 700941)	IV		Early	LG04-1459 x (DT97-290 (2) x PI 587982A)	Released by USDA, Agricultural Research Service
2	2	2019	10061-236-11		IV		Early	('5601T' x PI 587982A) x LG03-4561-14	
3	3	2019	10061-236-12	DS61-236	IV		Early	('5601T' x PI 587982A) x LG03-4561-14	
4	4	2019	10074-112		IV		Early	(DT97-4290 (2) x PI 587982A) x LG01-5087-5	
5	5	2019	11030-541-28	11030S	IV		Early	DS25-1 x PI 424324B	
6	6	2019	11043-224-91		IV		Early	DS25-1 x PI 417050	
7	7	2019	11043-225-72	DS43-72	IV	R	Early	DS25-1 x PI 417050	
8	8	2019	13082-1315		IV		Early	DS25-1 x (Bolivar x PI 567516C)	Experimental line
9	9	2019	65-414-132-1	DS65-1	III	R	Early	PI 587982A x S99-11509	
10	10	2019	CZ3841LL		III	S	Early		Commercial cultivar
11	11	2019	DB06x006-93	D06x006-93	III		Early	DT97-4290 x PI423941	Phomopsis resistant line, not released yet
12	12	2019	LD00-3309	PI 639740	IV	S	Early		Public cultivar
13	13	2019	LD06-7620		IV	S	Early		Public cultivar
14	14	2019	LG03-4561-14	DS03-14 (PI 689003)	III	S	Early	LG99-5106 x LG97-9226	Released by USDA, Agricultural Research Service
15	15	2019	Pella 86	PI 509044	III	S	Early		Public cultivar, release by Iowa State University in 1987
16	16	2019	PI 587982A		III	R	Early		
17	17	2019	PI 594619		III	R	Early		Hard seeded
18	18	2019	Progeny 4211		IV	S	Early		Commercial cultivar
19	19	2019	SS93-6181	PI 652443	IV	R	Early		Derived from PI 417479, released by University of Missouri-Columbia.
20	20	2019	10049-142-31	DS49-142 (PI 703498)	V	R	Late	(DT98-9102 x PI 603756) x 'Jake'	Released by USDA, Agricultural Research Service
21	21	2019	11030-242-22		IV		Late	DS25-1 x PI 424324B	
22	22	2019	11030-242-51		V		Late	DS25-1 x PI 424324B	
23	23	2019	11030-244-12		IV		Late	DS25-1 x PI 424324B	
24	24	2019	11030-244-41		IV		Late	DS25-1 x PI 424324B	
25	25	2019	11030-244-51		V		Late	DS25-1 x PI 424324B	
26	26	2019	11069-122-11		V		Late	DS25-1 x 'Osage'	
27	27	2019	11069-323-11		IV		Late	DS25-1 x 'Osage'	
28	28	2019	11069-333-11		V		Late	DS25-1 x 'Osage'	
29	29	2019	11069-512-34		V		Late	DS25-1 x 'Osage'	
30	30	2019	12047-1-160		V		Late	DS25-1 x DT97-4290	
31	31	2019	12047-1-178		V		Late	DS25-1 x DT97-4290	
32	32	2019	12060-260-2		IV		Late	(PI 587982A x DT97-4290) x 'LG00-3309'	
33	33	2019	12060-83-15		IV		Late	(PI 587982A x DT97-4290) x 'LG00-3309'	
34	34	2019	AG4632		IV	S	Late		Commercial cultivar
35	35	2019	AG51X8		V	S	Late		Commercial cultivar
36	36	2019	AG5335		V	S	Late		Commercial cultivar
37	37	2019	DA10x30-09F	USDA-ARS-GDA1030-9	IV		Late	DT99-17445 (PI 675997) x ‘J00-2’	Released by USDA, Agricultural Research Service
38	38	2019	DA10x30-48F		IV		Late		Experimental line
39	39	2019	DA1134-015F	D1134-015F	V		Late		Experimental line
40	40	2019	DA1221-01-597		IV		Late		Experimental line
41	41	2019	DA1239-09		IV		Late		Experimental line
42	42	2019	DA13062-004F	D13062004F	IV		Late		Experimental line
43	43	2019	DA13086-011F	D13086011F	IV		Late		Experimental line
44	44	2019	DA13086-048F	D13086048F	IV		Late		Experimental line
45	45	2019	DA13099-008F	D13099008F	V		Late		Experimental line
46	46	2019	DS25-1	PI 684675	IV	R	Late	DT98-9102 x PI 587982A	Released by USDA, Agricultural Research Service
47	47	2019	DT97-4290	PI 642055	IV	S	Late		Released by USDA, Agricultural Research Service
48	48	2019	Manokin	PI 559932	IV		Late		Public cultivar
49	49	2019	P46T59R		IV	S	Late		Commercial cultivar
50	50	2019	P48A32X		IV		Late		Commercial cultivar
51	51	2019	P48A60X		IV	S	Late		Commercial cultivar
52	1	2020	10031-243-12	DS31-243 (PI 700941)	IV		Early	LG04-1459 x (DT97-290 (2) x PI 587982A)	Released by USDA, Agricultural Research Service
53	2	2020	10061-236-12	DS61-236	IV		Early	('5601T' x PI 587982A) x LG03-4561-14	
54	3	2020	10074-112		IV		Early	(DT97-4290 (2) x PI 587982A) x LG01-5087-5	
55	4	2020	1130-851-111		III		Early	DS25-1 x PI 424324B	
56	5	2020	14119-211-10		IV	R	Early	(PI 587982A x S99-11509) x 'N8002'	Source of resistance is PI 587982A
57	6	2020	14119-211-24		IV		Early	(PI 587982A x S99-11509) x 'N8002'	Source of resistance is PI 587982A
58	7	2020	65-414-132-1	DS65-1	IV	R	Early	PI 587982A x S99-11509	
59	8	2020	Clark	PI 548533	IV		Early		Recurrent parent of 'Clark' maturity isolines
60	9	2020	CZ3841LL		III	S	Early		Commercial cultivar
61	10	2020	D06x006-93	DB06x006-93	IV		Early	DT97-4290 x PI 423941	Phomopsis resistant line, not released yet
62	11	2020	D13086011F	DA13086-011F	IV		Early		Experimental line
63	12	2020	D13086048F	DA13086-048F	IV		Early		Experimental line
64	13	2020	L63-2404	PI 547453	III		Early		Clark maturity isoline
65	14	2020	L63-3117	PI 547423	III		Early		Clark maturity isoline
66	15	2020	L66-432	PI 547432	IV		Early		Clark maturity isoline
67	16	2020	L74-441	PI 547610	IV		Early		Clark maturity isoline
68	17	2020	L80-5914	PI 547652	III		Early		Clark maturity isoline
69	18	2020	L94-1110	PI 591491	IV		Early		Clark maturity isoline
70	19	2020	LD06-7620		IV	S	Early		Public cultivar
71	20	2020	LG03-4561-14	DS03-14 (PI 689003)	III	S	Early	LG99-5106 x LG97-9226	Released by USDA, Agricultural Research Service
72	21	2020	P40A47X		IV		Early		Commercial cultivar
73	22	2020	Pella 86	PI 509044	III	S	Early		Public cultivar, release by Iowa State University in 1987
74	23	2020	Progeny 4211		IV	S	Early		Commercial cultivar
75	24	2020	SS93-6181	PI 652443	IV	R	Early		Derived from PI 417479, released by University of Missouri-Columbia.
76	25	2020	Y227-1		IV		Early	SS93-6181 x DT97-4290	
77	26	2020	10049-142-31	DS49-142 (PI 703498)	V	R	Late	(DT98-9102 x PI 603756) x 'Jake'	Released by USDA, Agricultural Research Service
78	27	2020	11069-122-11		V		Late	DS25-1 x 'Osage'	
79	28	2020	11069-323-11		IV		Late	DS25-1 x 'Osage'	
80	29	2020	11069-333-11		V		Late	DS25-1 x 'Osage'	
81	30	2020	12047-1-160		V		Late	DS25-1 x DT97-4290	
82	31	2020	12047-1-178		V		Late	DS25-1 x DT97-4290	
83	32	2020	12060-260-2		IV		Late	(PI 587982A x DT97-4290) x 'LG00-3309'	
84	33	2020	14119-721-61		V		Late	(PI 587982A x S99-11509) x N8002	Source of resistance is PI 587982A
85	34	2020	AG4632		IV	S	Late		Commercial cultivar
86	35	2020	AG5335		V	S	Late		Commercial cultivar
87	36	2020	D1134-015F	DA1134-015F	V		Late		Experimental line
88	37	2020	D13062-001	DA13062-001F	V		Late		Experimental line
89	38	2020	D13062004F	DA13062-004F	IV		Late		Experimental line

90	39	2020	D13062-015	DA13062-015	IV		Late		Experimental line
91	40	2020	D13076-042	DA13076-042	IV		Late		Experimental line
92	41	2020	D13092-015	DA13092-015F	IV		Late		Experimental line
93	42	2020	D13092-039	DA13092-039F	IV		Late		Experimental line
94	43	2020	D13099008F	DA13099-008F	IV		Late		Experimental line
95	44	2020	D1486-10	DA1486-10	IV		Late		Experimental line
96	45	2020	D1488-0228	DA1488-0228F	IV		Late		Experimental line
97	46	2020	DA15-x085-54		IV		Late	DS25-1 x DA14c034-03-14	In both E & L Test, High oleic acid germplasm line.
98	47	2020	DS25-1	PI 684675	IV	R	Late	DT98-9102 x PI 587982A	Released by USDA, Agricultural Research Service
99	48	2020	L65-3336		IV		Late		Clark maturity isoline
100	49	2020	L74-441	PI 547610	IV		Late		Clark maturity isoline
101	50	2020	L92-1195	PI 591490	IV		Late		Clark maturity isoline
102	51	2020	Manokin	PI 559932	IV		Late		Public cultivar
103	52	2020	P46T59R		IV	S	Late		Commercial cultivar
104	53	2020	P48A60X		IV	S	Late		Commercial cultivar
105	1	2021	10031-243-12	DS31-243 (PI 700941)	IV		Early	LG04-1459 x (DT97-290 (2) x PI 587982A)	Released by USDA, Agricultural Research Service
106	2	2021	10074-112		IV		Early	(DT97-4290 (2) x PI 587982A) x LG01-5087-5	
107	3	2021	1130-528-151		IV		Early	DS25-1 x PI 424324B	
108	4	2021	14119-211-10		IV	R	Early	(PI 587982A x S99-11509) x 'N8002'	Source of resistance is PI 587982A
109	5	2021	1419-211-521		IV		Early	(PI 587982A x S99-11509) x 'N8002'	
110	6	2021	15087-511-46		IV		Early	(PI 587982A x N98-4445A) x LG10-3671-1	
111	7	2021	15087-511-51		IV		Early	(PI 587982A x N98-4445A) x LG10-3671-1	
112	8	2021	15097-225-21		IV		Early		Experimental line
113	9	2021	16028-35-31		IV		Early	DS25-1 x (PI 594445 x 'Osage'	
114	10	2021	65-414-132-1	DS65-1	IV	R	Early	PI 587982A x S99-11509	
115	11	2021	Clark	PI 548533	IV		Early		Recurrent parent of 'Clark' maturity isolines
116	12	2021	CZ3841LL		III	S	Early		Commercial cultivar
117	13	2021	DA15-x085-54		IV		Early	DS25-1 x DA14c034-03-14	In both E & L Test, High oleic acid germplasm line.
118	14	2021	DA1602-08-B7		IV		Early	DS25-1 x DA08x43-33F	
119	15	2021	DA1602-13-B7		IV		Early	DS25-1 x DA08x43-33F	
120	16	2021	DA1602-14-B7		IV		Early	DS25-1 x DA08x43-33F	
121	17	2021	DA1602-15-B7		III		Early	DS25-1 x DA08x43-33F	
122	18	2021	DA1602-16-B7		IV		Early	DS25-1 x DA08x43-33F	
123	19	2021	L63-2404	PI 547453	III		Early		Clark maturity isoline
124	20	2021	L63-3117	PI 547423	III		Early		Clark maturity isoline
125	21	2021	L66-432	PI 547432	IV		Early		Clark maturity isoline
126	22	2021	L74-441	PI 547610	IV		Early		Clark maturity isoline
127	23	2021	L80-5914	PI 547652	III		Early		Clark maturity isoline
128	24	2021	L94-1110	PI 591491	IV		Early		Clark maturity isoline
129	25	2021	LD00-3309	PI 639740	IV	S	Early		Public cultivar
130	26	2021	LG03-4561-14	DS03-14 (PI 689003)	III	S	Early	LG99-5106 x LG97-9226	Released by USDA, Agricultural Research Service
131	27	2021	Progeny 4211		IV	S	Early		Commercial cultivar
132	28	2021	SS93-6181	PI 652443	IV	R	Early		Derived from PI 417479, released by University of Missouri-Columbia.
133	29	2021	10049-142-31	DS49-142 (PI 703498)	V	R	Late	(DT98-9102 x PI 603756) x 'Jake'	Released by USDA, Agricultural Research Service
134	30	2021	11069-323-11		IV		Late	DS25-1 x 'Osage'	
135	31	2021	12060-260-2		IV		Late	(PI 587982A x 'DT97-4290') x 'LG00-3309'	
136	32	2021	1335-331-422		V		Late	PI 594619 x LD06-7620	
137	33	2021	AG4632		IV	S	Late		Commercial cultivar
138	34	2021	AG5335		V	S	Late		Commercial cultivar
139	35	2021	AG55X7		V		Late		Commercial cultivar
140	36	2021	D1405-212-3F	DA1405-212-3F	V		Late		Experimental line
141	37	2021	D1405-221-1F	DA1405-221-1F	V		Late		Experimental line
142	38	2021	D1488-0228	DA1488-0228F	IV		Late		Experimental line
143	39	2021	DA1134-015F	D1134-015F	V		Late		Experimental line
144	40	2021	DA13062-001F	D13062-001	V		Late		Experimental line
145	41	2021	DA13092-015F	D13092-015	IV		Late		Experimental line
146	42	2021	DA13092-039F	D13092-039	IV		Late		Experimental line
147	43	2021	DA13099-008F	D13099008F	V		Late		Experimental line
148	44	2021	DA1539-109F		IV		Late		Experimental line
149	45	2021	DA1541-102F		IV		Late		Experimental line
150	46	2021	DA1568-112F		V		Late		Experimental line
151	47	2021	DA1585-564F		V		Late		Experimental line
152	48	2021	DA15-x085-54		IV		Late	DS25-1 x DA14c034-03-14	In both E & L Test, High oleic acid germplasm line.
153	49	2021	DS25-1	PI 684675	IV	R	Late	DT98-9102 x PI 587982A	Released by USDA, Agricultural Research Service
154	50	2021	L65-3366		IV		Late		Clark maturity isoline
155	51	2021	L92-1195	PI 591490	IV		Late		Clark maturity isoline
156	52	2021	P46T59R		IV	S	Late		Commercial cultivar
157	53	2021	P48A60X		IV	S	Late		Commercial cultivar
158	1	2022	10031-243-12	DS31-243 (PI 700941)	IV		Early	LG04-1459 x (DT97-290 (2) x PI 587982A)	Released by USDA, Agricultural Research Service
159	2	2022	13099-263-11		IV		Early	D49-2491 (6) x 'Hawkeye'	D49-2491 is the recurrent parent of a maturity isoline
160	3	2022	14119-211-10		IV	R	Early	(PI 587982A x S99-11509) x 'N8002'	Source of resistance is PI 587982A
161	4	2022	1587-513-913		IV		Early	(PI 587982A x N98-4445A) x LG10-3671-1	
162	5	2022	1587-513-922		IV		Early	(PI 587982A x N98-4445A) x LG10-3671-1	
163	6	2022	16016-114-21		IV		Early	DT97-4290 (3) x PI 587982A	
164	7	2022	16023-617-32		IV		Early	(LG01-5087 x DT97-4290) x (PI 594619 x LG01-5087-5)	Parent PI 594619 is hardseeded (impermeable seed coat).
165	8	2022	16031-131-12		IV		Early	(PI 587982A x DT97-4290) x ('Stoddard' x LG01-5087-5)	
166	9	2022	16031-131-2		IV		Early	(PI 587982A x DT97-4290) x ('Stoddard' x LG01-5087-5)	
167	10	2022	65-414-132-1	DS65-1	IV	R	Early	PI 587982A x S99-11509	
168	11	2022	Clark	PI 548533	IV		Early		Recurrent parent of 'Clark' maturity isolines
169	12	2022	DA1570-25F		IV		Early	DB06x006-169 x DB06x006-93	Derived from PI 423941
170	13	2022	DA1570-29F		IV		Early	DB06x006-169 x DB06x006-93	Derived from PI 423941
171	14	2022	DA1571-10F		IV		Early	DB06x016-142 x DB06x006-93	Derived from PI 423941
172	15	2022	DA1571-18F		IV		Early	DB06x016-142 x DB06x006-93	Derived from PI 423941
173	16	2022	DA1572-403F		IV		Early	DB06x016-142 x DB06x006-161	Derived from PI 423941
174	17	2022	DA1579-08F		IV		Early	DB06x016-142 x SS93-6181	Derived from PI 423941 and SS93-6181
175	18	2022	L63-2404	PI 547453	III		Early		Clark maturity isoline
176	19	2022	L63-3117	PI 547423	III		Early		Clark maturity isoline
177	20	2022	L66-432	PI 547432	IV		Early		Clark maturity isoline
178	21	2022	L74-441	PI 547610	IV		Early		Clark maturity isoline
179	22	2022	L80-5914	PI 547652	III		Early		Clark maturity isoline
180	23	2022	L94-1110	PI 591491	IV		Early		Clark maturity isoline
181	24	2022	LD00-3309	PI 639740	IV	S	Early		Public cultivar
182	25	2022	LG03-4561-14	DS03-14 (PI 689003)	III	S	Early	LG99-5106 x LG97-9226	Released by USDA, Agricultural Research Service

183	26	2022	Progeny 4211		IV	S	Early		Commercial cultivar
184	27	2022	SS93-6181	PI 652443	IV	R	Early		Derived from PI 417479, released by University of Missouri-Columbia.
185	28	2022	10049-142-31	DS49-142 (PI 703498)	V	R	Late	(DT98-9102 x PI 603756) x 'Jake'	Released by USDA, Agricultural Research Service
186	29	2022	1121-121-111		V		Late	D49-2491 (6) x 'Hawkeye'	D49-2491 is the recurrent parent of a maturity isoline
187	30	2022	12060-260-2		IV		Late	(PI 587982A x DT97-4290) x 'LG00-3309'	
188	31	2022	13072-293-11		IV		Late	DS25-1 x PI424546A	
189	32	2022	13072-293-12		IV		Late	DS25-1 x PI424546A	
190	33	2022	16023-616-42		IV		Late	(LG01-5087 x DT97-4290) x (PI 594619 x LG01-5087-5)	Parent PI 594619 is hardseeded.
191	34	2022	16027-312-12		IV		Late	DS25-1 x ((PI 594445 x 'Jake'))	
192	35	2022	AG5335		V	S	Late		Commercial cultivar
193	36	2022	AG55X7		V		Late		Commercial cultivar
194	37	2022	D49-2491	PI 548980	VI		Late		Maturity isoline, sister line of 'Lee' and recurrent parent for isoline set
195	38	2022	DA10x30-09F	USDA-ARS-GDA1030-9	IV		Late	DT99-17445 (PI 675997) x 'J00-2'	Released by USDA, Agricultural Research Service
196	39	2022	DA1134-015F	D1134-015F	V		Late		Experimental line
197	40	2022	DA13092-015F	D13092-015	IV		Late		Experimental line
198	41	2022	DA13099-008F	D13099008F	V		Late		Experimental line
199	42	2022	DA1488-0228F	D1488-0228	IV		Late		Experimental line
200	43	2022	DA1539-1010F		V		Late		Experimental line
201	44	2022	DA1539-109F		IV		Late		Experimental line
202	45	2022	DA1541-102F		IV		Late		Experimental line
203	46	2022	DA1601-18		IV		Late		Experimental line
204	47	2022	DS25-1	PI 684675	IV	R	Late	DT98-9102 x PI 587982A	Released by USDA, Agricultural Research Service
205	48	2022	Ellis	PI 680630	IV		Late		Public cultivar
206	49	2022	Hood	PI 548980	VI		Late		Public cultivar
207	50	2022	L65-3366		IV		Late		Clark maturity isoline
208	51	2022	L92-1195	PI 591490	IV		Late		Clark maturity isoline
209	52	2022	P46T59R		IV	S	Late		Commercial cultivar
210	53	2022	P48A60X		IV	S	Late		Commercial cultivar
211	1	2023	11043-225-72	DS43-72	IV	R	Early	DS25-1 x PI 417050	
212	2	2023	14119-211-10		IV	R	Early	(PI 587982A x S99-11509) x 'N8002'	Source of resistance is PI 587982A
213	3	2023	16023-617-32		IV		Early	(LG01-5087 x DT97-4290) x (PI 594619 x LG01-5087-5)	Parent PI 594619 is hardseeded (impermeable seed coat).
214	4	2023	1623-516-141		IV		Early	(LG01-5087 x DT97-4290) x (PI 594619 x LG01-5087-5)	Parent PI 594619 is hardseeded (impermeable seed coat).
215	5	2023	1623-516-143		IV		Early	(LG01-5087 x DT97-4290) x (PI 594619 x LG01-5087-5)	Parent PI 594619 is hardseeded (impermeable seed coat).
216	6	2023	1623-516-151		IV		Early	(LG01-5087 x DT97-4290) x (PI 594619 x LG01-5087-5)	Parent PI 594619 is hardseeded (impermeable seed coat).
217	7	2023	18011-53-21		IV		Early	SS93-6181 x 10031-243-12	
218	8	2023	18011-53-41		IV		Early	SS93-6181 x 10031-243-12	
219	9	2023	18016-41-21		IV		Early	10031-243-12 x (SS93-6181 x DT97-4290)	
220	10	2023	18019-45-23		IV		Early	10061-236-11 x 10031-243-12	
221	11	2023	18019-45-31		IV		Early	10061-236-11 x 10031-243-12	
222	12	2023	18019-45-32		IV		Early	10061-236-11 x 10031-243-12	
223	13	2023	65-414-132-1	DS65-1	IV	R	Early	PI 587982A x S99-11509	
224	14	2023	Clark	PI 548533	IV		Early		Recurrent parent of 'Clark' maturity isolines
225	15	2023	DS34-1		IV		Early	PI 587982A x DT97-4290	Released by USDA, Agricultural Research Service
226	16	2023	L63-2404	PI 547453	III		Early		Clark maturity isoline
227	17	2023	L66-432	PI 547432	IV		Early		Clark maturity isoline
228	18	2023	LD00-3309	PI 639740	IV	S	Early		Public cultivar
229	19	2023	LG03-4561-14	DS03-14 (PI 689003)	III	S	Early	LG99-5106 x LG97-9226	Released by USDA, Agricultural Research Service
230	20	2023	Pella 86	PI 509044	III	S	Early		Public cultivar, release by Iowa State University in 1987
231	21	2023	PI 587982A		III	R	Early		
232	22	2023	Progeny 4211		IV	S	Early		Commercial cultivar
233	23	2023	SS93-6181	PI 652443	IV	R	Early		Derived from PI 417479, released by University of Missouri-Columbia.
234	24	2023	10049-142-31	DS49-142 (PI 703498)	V	R	Late	(DT98-9102 x PI 603756) x 'Jake'	Released by USDA, Agricultural Research Service
235	25	2023	12060-260-2		IV		Late	(PI 587982A x DT97-4290) x 'LG00-3309'	
236	26	2023	1587-513-922		IV		Late	(PI 587982A x N98-4445A) x LG10-3671-1	
237	27	2023	16016-114-21		IV		Late	DT97-4290 (3) x PI 587982A	
238	28	2023	16018-144-21		IV		Late	DT97-4290 (5) x L91-8060	Stem termination isoline derived from DT97-4290
239	29	2023	16019-224-11		IV		Late	DT97-4290 (5) x L62-1251	Stem termination isoline derived from DT97-4290
240	30	2023	16020-111-22		V		Late	DS-880 (5) x L91-8060	Stem termination isoline derived from DS-880
241	31	2023	16021-234-11		V		Late	DS-880 (5) x L62-1251	Stem termination isoline derived from DS-880
242	32	2023	16022-113-23		V		Late	DS-880 (5) x 'Clark'	Stem termination isoline derived from DS-880
243	33	2023	16023-616-42		IV		Late	(LG01-5087 x DT97-4290) x (PI 594619 x LG01-5087-5)	Parent PI 594619 is hardseeded.
244	34	2023	16027-312-12		IV		Late	DS25-1 x ((PI 594445 x 'Jake'))	
245	35	2023	17002-14-51		IV		Late	DT97-4290 (5) x L63-3297	Stem termination isoline derived from DT97-4290
246	36	2023	18019-45-33		IV		Late	10061-236-11 x 10031-243-12	
247	37	2023	AG5335		V	S	Late		Commercial cultivar
248	38	2023	AG55X7		V		Late		Commercial cultivar
249	39	2023	DA13099-008F	D13099008F	V		Late		Experimental line
250	40	2023	DA1539-1010F		V		Late		Experimental line
251	41	2023	DA1541-102F		IV		Late		Experimental line
252	42	2023	DA1570-25F		IV		Late	DB06x006-169 x DB06x006-93	Derived from PI 423941
253	43	2023	DA1571-10F		IV		Late	DB06x016-142 x DB06x006-93	Derived from PI 423941
254	44	2023	DA1571-16F		IV		Late	DB06x016-142 x DB06x006-93	Derived from PI 423941
255	45	2023	DA1571-38F		IV		Late	DB06x016-142 x DB06x006-93	Derived from PI 423941
256	46	2023	DA1579-04F		IV		Late	DB06x016-142 x SS93-6181	Derived from PI 423941 and SS93-6181
257	47	2023	DA1579-08F		IV		Late	DB06x016-142 x SS93-6181	Derived from PI 423941 and SS93-6181
258	48	2023	DA1601-18		IV		Late		Experimental line
259	49	2023	DS25-1	PI 684675	IV	R	Late	DT98-9102 x PI 587982A	Released by USDA, Agricultural Research Service
260	50	2023	DS-880	PI 659348	V		Late		Released by USDA, Agricultural Research Service
261	51	2023	DT97-4290	PI 642055	IV	S	Late		Released by USDA, Agricultural Research Service
262	52	2023	L65-3366		IV		Late		Clark maturity isoline
263	53	2023	L74-441	PI 547610	IV		Late		Clark maturity isoline
264	54	2023	L92-1195	PI 591490	IV		Late		Clark maturity isoline
265	55	2023	P46T59R		IV	S	Late		Commercial cultivar
266	56	2023	P48A60X		IV	S	Late		Commercial cultivar

* Name of seed samples obtained from breeders for assay each year

** Maturity group

*** R: Resistant check; S: Susceptible check.

**** Early: tested soybean entries from maturity group (MG) III through early MG IV; Late: tested soybean entries from MG IV through early MG V.
