# Supplementary material for: Multi-year evaluation and release of improved soybean lines for resistance to Phomopsis seed decay
Source: PLoS One. 2026 Aug 24;21(8):e0354166. doi: 10.1371/journal.pone.0354166 (PMC13502683; doi:10.1371/journal.pone.0354166)
Supplement: Supplementary file 2 [file pone.0354166.s002.docx]

**S1 Fig.**  **Average daily maximum temperatures, average daily maximum relative humidity, total precipitation and number of rainy days during the soybean growing seasons for the months of April through October** **from 2019 to 2023 at Stoneville, Mississippi**
